# EBNA1 inhibitors reveal CDC7 and POU2F1 as direct functional targets in EBV epithelial cancers

**DOI:** 10.1128/mbio.00329-26

**Published:** 2026-05-14

**Authors:** Songtao He, Niseno Terhuja, Samantha S. Soldan, Christopher Chen, Joel Cassel, Xiangfan Yin, Qin Liu, Sun Sook Chung, Leonardo Josué Castro-Muñoz, Leena Yoon, Jie Wang, Joseph M. Salvino, Benjamin E. Gewurz, Italo Tempera, Troy E. Messick, Paul M. Lieberman

**Affiliations:** 1 The Wistar Institute 36586, Philadelphia, Pennsylvania, USA; 2 Saint Joseph's University, Philadelphia, Pennsylvania, USA; 3 Brigham and Women’s Hospital, Harvard Medical School 1811 , Boston, Massachusetts, USA; Princeton University, Princeton, New Jersey, USA

**Keywords:** EBV, EBNA1, inhibitors, epithelial cancers, CDC7, POU2F1

## Abstract

Epstein-Barr virus (EBV) latent infection is causally linked to several epithelial cancers, including endemic forms of undifferentiated nasopharyngeal carcinoma (NPC), and to a subtype of gastric cancer (GC). EBNA1 is the virus-encoded sequence-specific DNA-binding protein required for episome maintenance but also contributes to host-cell survival through multiple mechanisms, including binding to the host chromosome. We previously developed small-molecule inhibitors of EBNA1 DNA-binding that block host cell cycle progression and growth of EBV+ cell lines and tumor models *in vivo*. However, the underlying molecular mechanisms of EBNA1 function and inhibition have not been completely elucidated. In this study, we employ VK1727 to inhibit EBNA1 DNA-binding to viral and cellular genomes in three EBV+ epithelial tumor-derived cell models (patient-derived xenograft C15, C666-1, and SNU719). We integrate EBNA1 ChIP-seq and transcriptomic RNA-seq analyses to identify the cell cycle-dependent kinase CDC7 and a stem cell transcription factor POU2F1 as direct functional targets of EBNA1 in each of these epithelial cancer models. EBNA1 binding to the CDC7 promoter and POU2F1 intron promotes RNA Pol II-pS5 to initiate transcription of these two genes. We show that CDC7 inhibitor simurosertib phenocopies VK1727, while Bcl-2 inhibitor venetoclax shows a synergistic effect with VK1727 for inhibition of EBV+ epithelial cancer cell proliferation and survival. Our study reveals new functional gene targets and pathways of VK1727 in EBV+ epithelial cancers that provide new biomarkers and combinatorial strategies to treat EBV-driven cancers.

**IMPORTANCE:**

EBNA1 is essential for Epstein-Barr virus (EBV) latency and tumorigenesis, but its mechanism of action on host gene expression is not yet known. Small-molecule inhibitors of EBNA1 DNA-binding block cell cycle progression and inhibit the growth of EBV+ tumors. In this study, we use the EBNA1 small-molecule inhibitor VK1727 to identify cellular gene targets that are bound by EBNA1 and deregulated by its pharmacological inhibition in EBV+ epithelial cancer cell lines and an NPC patient-derived xenograft mouse model. We identify cell cycle-dependent kinase CDC7 and the stem cell transcription factor POU2F1 as EBNA1-bound and regulated genes important for EBV epithelial cancer proliferation. These findings not only decipher the molecular mechanism by which VK1727 blocks cell cycle progression and inhibits cell proliferation but also provide two new cellular gene targets and pathways for therapeutic intervention in EBV+ epithelial cancers.

## INTRODUCTION

Epstein-Barr virus (EBV) is a human gammaherpesvirus that establishes lifelong latent infection in more than 95% of the human population (1). EBV is also classified as a tumor virus due to its strong association with several human cancers, including Burkitt’s lymphoma (BL), Hodgkin’s disease (HD), gastric (GC), and nasopharyngeal carcinoma (NPC) (2). EBV epithelial cancers represent ~70% of all EBV-associated cancers worldwide (3). NPC is highly prevalent in southern China, Southeast Asia, and some North African countries, with an incidence of 4–25 cases per 100,000 individuals (4). EBV-associated gastric cancer (EBVaGC) is a specific molecular subtype comprising ~10% of all gastric cancers worldwide (5). EBV DNA and viral gene products are readily detected in EBV epithelial cancers, but the patterns of gene expression and the targets of oncogenic transformation in epithelial tumors are generally different from those in EBV-associated lymphomas. While EBV can efficiently immortalize B-lymphocytes in cell culture and cause B-cell lymphomas in immunosuppressed individuals (6, 7), its mechanism of oncogenic transformation in epithelial cancers is less well understood.

EBV infection of normal epithelial cells results in a transient and lytic replication cycle (8). In EBV epithelial cancers, EBV maintains a latent state that retains episomal DNA in tumor cells (9, 10). Most epithelial cancers express a type II latency, characterized by the limited expression of only EBNA1 and LMP1/2 proteins, and many non-coding RNAs, including EBERs and BARTs (11). LMP1 is a well-characterized viral oncogene that functions as a viral mimic of the TNFR family member, CD40, that activates signaling pathways related to cellular growth and survival (12). LMP2 mimics BCR signaling in B-lymphocytes and related Src-family kinase pathways in epithelial cells (13, 14). The EBV non-coding RNAs, including many miRNAs, are known to have diverse activities that could contribute to viral oncogenesis (15). EBNA1 is ubiquitously expressed in most EBV-positive tumor cells in NPC and GC (16). Numerous studies have described the versatility of EBNA1 in maintaining episome structure, promoting viral replication, tethering episomes to metaphase chromosomes, which is essential for maintaining EBV latency, regulating host gene expression, and interacting with host proteins involved in viral sensing and cancer signaling pathways (16–18). Increasing evidence reveals an oncogenic role of EBNA1 in EBV-associated epithelial cancers, such as promoting the epithelial-mesenchymal transition (EMT) process (19), inducing genomic instability (20), and enhancing immune evasion (21). While EBNA1 has been implicated in each of these oncogenic pathways, the specific molecular mechanisms require further investigation.

In addition to its direct binding to the EBV episome, EBNA1 can also bind to many sites in the host genome (22, 23). Previous studies from our lab characterized some EBNA1-bound gene targets in the cellular genome, such as MEF2B, EBF1, and IL6R in BL cell lines (24), and gastrokines GKN1 and GKN2 in EBVaGC cell lines (25). Others have found EBNA1 regulates NOX2 in EBVaGC cell lines to modulate ROS (26) and induce telomere dysfunction (27), and BMP2 in NPC (28). EBNA1 can also activate viral genes, such as EBNA2, in type III latency (29), and this is thought to be mediated by long-distance enhancer-like DNA-loop interactions and other epigenetic effects (30–32). However, the mechanism of cellular gene regulation by EBNA1 remains poorly understood compared to the more potent superenhancer formation by EBNA2 and EBNA3C (33, 34).

EBNA1 has been considered an attractive target for small-molecule inhibition due to its essential role in maintaining EBV episomes during latency, its consistent expression in cancer cells, and its unique and druggable protein structure (35, 36). A series of small-molecule inhibitors has been developed using a structure-based drug design approach and shown to bind directly to EBNA1 and sterically inhibit DNA binding (36). VK1727 has been shown to reduce EBNA1 DNA-binding to oriP DNA *in vivo* and reduce viral copy number in EBV-associated tumors (37). A very closely related analog to VK1727 (namely VK2019) has been investigated in a clinical trial to treat NPC (38). While on-target engagement of VK2019 was detected in tumor biopsies, clinical response was limited. Both VK1727 and VK2019 reduce EBV persistence in epithelial cancer cells and prevent tumor growth in mouse models of NPC and EBVaGC *in vivo* (37, 38). In addition, our lab demonstrated VK1727 specifically blocks cell cycle progression of EBV-associated tumor-derived cell lines and mouse xenograft models (37). In this work, we investigate the mechanism through which the EBNA1 inhibitor VK1727 blocks cell cycle progression of EBV-associated epithelial cancer cells. We assayed the effect of VK1727 on the genome-wide DNA binding of EBNA1 by ChIP-seq, and the transcriptional response by RNA-seq in EBVaGC line SNU719, EBV+ NPC cell line C666-1, and in the C15 patient-derived xenograft (PDX) mouse model of NPC. We integrated these data sets to identify common gene targets of VK1727. We further characterized two functional gene targets of EBNA1 (CDC7 and POU2F1) in EBV+ epithelial cancers, which are involved in cell cycle progression and cell fate determination. Our results here provide two potential biomarkers for evaluating the therapeutic activity of EBNA1 inhibitors in EBV+ epithelial cancers.

## RESULTS

### EBNA1 inhibitors target cell cycle control in three different EBV tumor models

**Fig 1:**
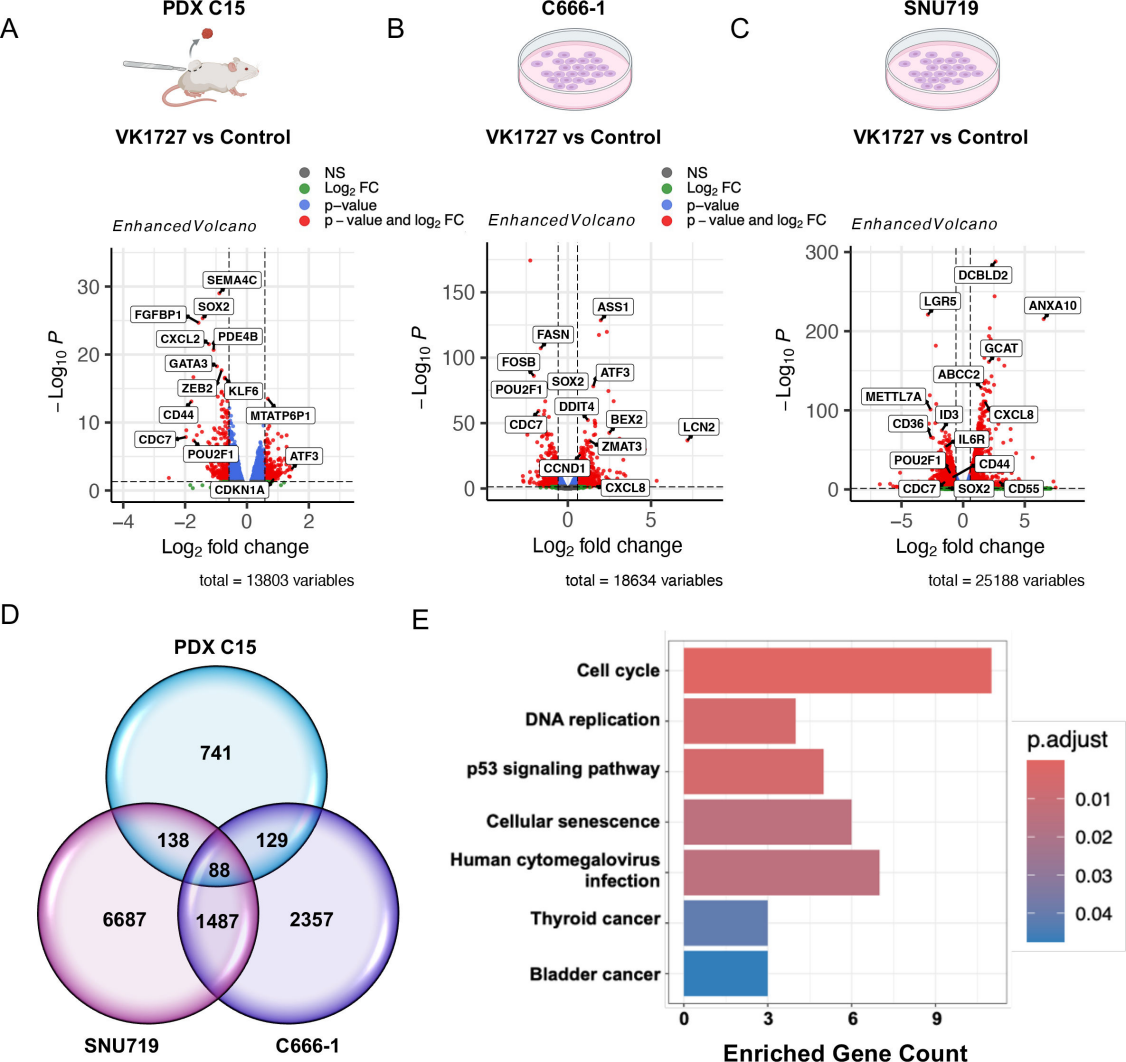
RNAseq analysis of VK1727 in three different EBV tumor models. (**A**) Volcano plot of differentially expressed cellular genes in VK1727-treated PDX C15. In all, 1,096 differentially expressed genes were identified with statistical significance (*P* value ≤ 0.05). Red dots represent differentially expressed genes with absolute value of fold change ≥ 0.58 and *P* value ≤ 0.05, green dots represent absolute value of fold change > 0.58 but *P* value > 0.05, blue dots represent absolute value of fold change < 0.58 but *P* value < 0.05 and black dots are genes that do not have significant expression change, with fold change < 0.58 and *P* value > 0.05. *X*-axis numerical scale labels are 2-base logarithm of fold change. *Y*-axis numerical scale labels are the minus form of the 10-base logarithm of the *P*-value. (**B**) Volcano plot of differentially expressed cellular genes in VK1727-treated C666-1 cells. RNA-seq analysis characterized 4,061 differentially expressed genes with statistical significance (*P* value ≤ 0.05). (**C**) Volcano plot of differentially expressed cellular genes in VK1727-treated SNU719 cells. RNA-seq analysis of VK1727 SNU719 cells identified 8,400 differentially expressed genes with statistical significance (*P* value ≤ 0.05). (**D**) Venn diagram of overlap genes among three VK1727-treated tumor models. Differentially expressed genes were enriched with statistical significance (*P* value ≤ 0.05) in each VK1727 treatment. The BioVenn was used to enrich the overlap of downregulated and upregulated genes among three tumor models. (**E**) Function analysis of overlap genes among three VK1727-treated tumor models. The clusterProfiler package was employed to annotate 88 overlap genes into the Kyoto Encyclopedia of Genes and Genomes (KEGG) database. DOSE and ggplot2 were used to generate a dot plot of enriched signaling pathways. Count denotes enriched gene count in each signaling pathway. Adjusted *P* value was employed to assess the statistical significance of an enriched signaling pathway.

Our lab previously reported that EBNA1 inhibitor VK1727 selectively blocks cell cycle progression and tumor growth of EBV+ epithelial cancer cell models (37). But the underlying molecular mechanism remains unclear. To identify direct effects of EBNA1 inhibition, we treated three EBV tumor-derived cell models with VK1727 for relatively short durations (PDX C15, 5 days; C666-1, 2 days; SNU719, 2 days) and performed RNA-seq analysis. C15 is an EBV-associated NPC patient-derived xenograft model that was transplanted into NOD mice (NOD.*Cg-Prkdc^scid^ Il2rgtm1Wjl/ScJ*) for sustaining tumor growth (36). C666-1 cell line is derived from an EBV-associated NPC patient and maintains EBV type II/I latency (39). SNU719 cell line is derived from EBV-associated gastric carcinoma and consistently maintains EBV persistence (40). Transcriptomic analysis identified differentially expressed genes after VK1727 treatments for each cell line or PDX (Fig. 1A through D). For PDX C15, there were 1,096 differentially expressed genes after VK1727 treatment (*P* < 0.05; Fig. 1A and D; Data File S1). The top four enriched signaling pathways included cell cycle, TNF signaling pathway, cellular senescence, and P53 signaling pathway (*P* value < 0.05; Fig. S1A). Cell cycle was ranked as the top one signaling pathway, with an enrichment score of 5.463 at *P* < 0.001 (Fig. S1B). Transcriptomic and functional analysis of VK1727-treated C666-1 cells identified 4,061 differentially expressed genes, primarily associated with P53 signaling pathway, cell cycle regulation, cellular senescence, and TNF signaling (*P* value < 0.05; Fig. 1B and D; Fig. S2A and B; Data File S1). The P53 signaling and cell cycle pathways were identified in the top six most significantly altered signaling pathways (*P* < 0.0008; Fig. S2B). RNA-seq analysis of VK1727-treated SNU719 cells revealed 8,400 differentially expressed genes, enriched in cell cycle regulation and P53 signaling pathway, as well as Ferroptosis and PPAR signaling pathway (*P* < 0.05; Fig. 1C and D; Fig. S3A and B; Data File S1). Cell cycle was listed as the most significantly altered signaling pathway with a *z*-score of 6.0708 at *P* < 0.001 (Fig. S3B). After that, we performed an overlap analysis of differentially expressed genes across three VK1727-treated tumor-derived cell models. In all, 88 overlaps of differentially expressed genes were enriched across three VK1727 treatments (Fig. 1D), with overlaps of 35 upregulated (Fig. S4A) and 53 downregulated (Fig. S4B) genes. Functional analysis demonstrated that these 88 overlap genes were enriched for cell cycle, DNA replication, P53 signaling pathway, and cellular senescence (*P* value < 0.05; Fig. 1E; Table S1).

We also performed differential expression analysis of EBV genes across the three tumor models (Fig. S5). In VK1727-treated PDX C15 tumors, only a few viral genes were affected, and the observed fold change was modest (<2-fold) (Fig. S5, left column). These include upregulation of non-coding RNA EBER2 and RPMS1, and downregulation of EBNA1, LMP1, EBER1, and BNRF1 (*P* < 0.01). VK1727-treated C666-1 also showed modest fold changes (<2-fold) for 11 genes, seven upregulated (EBER2, RPMS1, BILF1, BALF2, BARF1, BHLF1, and BMRF2) and four downregulated genes (EBNA1, LMP1, EBER1, and EBNA3A) (Fig. S5, middle column). VK1727-treated SNU719 cells affected 22 EBV genes including upregulation of EBER1 and 2, RPMS1, LF1, LF2, LF3, and downregulation of EBNA1, LMP2A, BRLF1, BMRF1, and BPLF1 (Fig. S5, right column). Across all three cell models, only EBER-2 and RPMS1 were consistently upregulated, and EBNA-1 was consistently downregulated, while EBNA1 was consistently upregulated. These findings indicate that VK1727 has modest effects on EBV gene transcript levels at these early time points after treatment, and that different cell types have different viral gene responses to EBNA1 inhibition.

### Integration of EBNA1 ChIP-seq with VK1727 RNA-seq to identify direct targets of EBNA1

**Fig 2:**
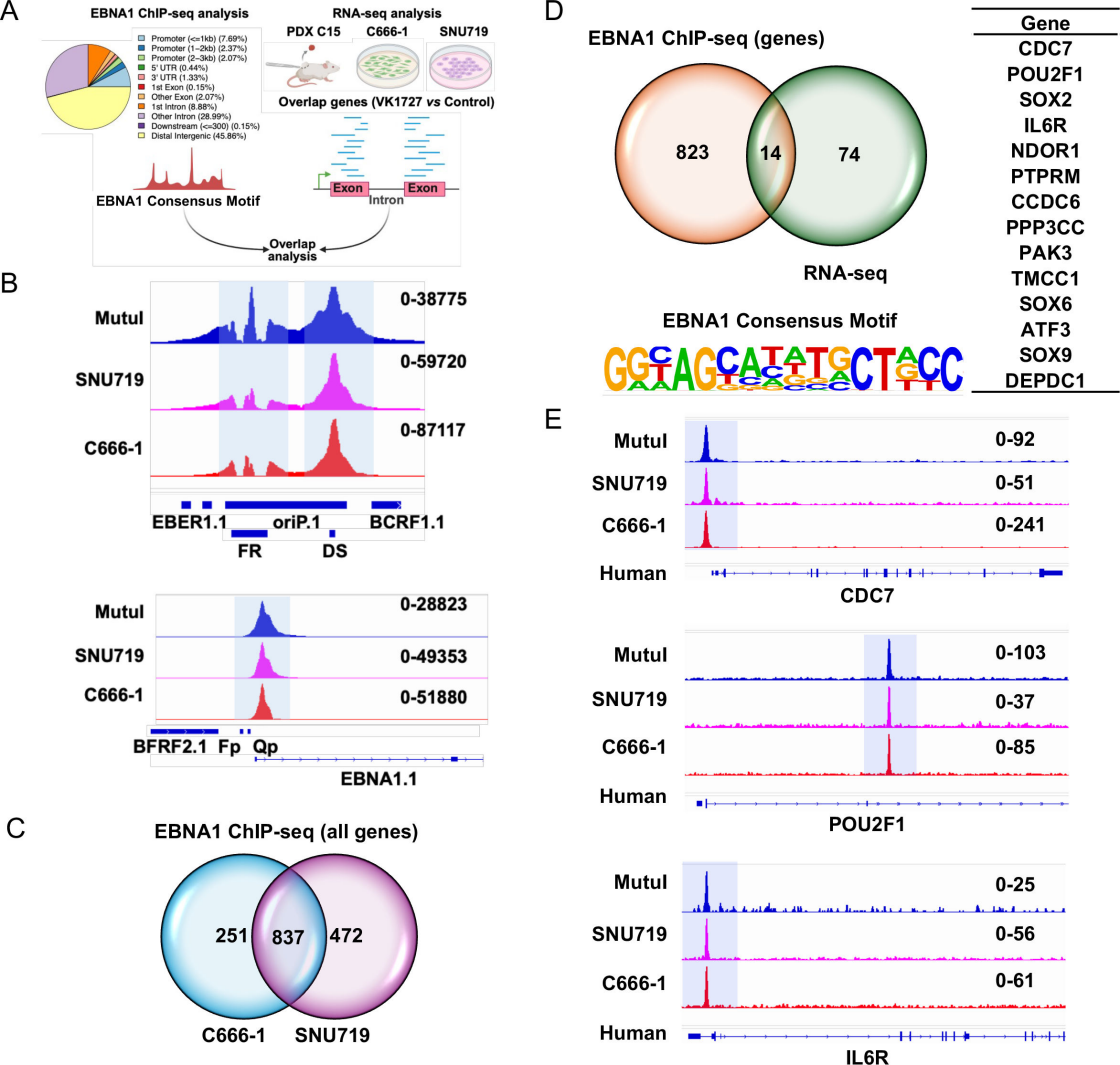
Integrated analysis of EBNA1 ChIP-seq with VK1727 RNAseq. (**A**) Schematic diagram for the identification of VK1727 targets in differentially expressed gene sets among three tumor models. Potential targets of VK1727 were identified using consensus EBNA1-binding sites in viral and cellular genomes in SNU719 and C666-1 cells. Then, an overlap analysis was performed to identify differentially expressed genes near EBNA1-binding sites, which was achieved using the match function in the R program. (**B**) EBNA1 ChIP-seq analysis of viral genomes in Mutu1, SNU719, and C666-1 cells. Consensus EBNA1-binding sites were characterized in FR, DS, and Qp regions in viral genomes in Mutu1, C666-1, and SNU719 cells. (**C**) EBNA1 conservative binding sites in cellular genomes between C666-1 and SNU719 cell lines. The bedtools were used to identify EBNA1 consensus binding sites between these two cell lines. In all, 1,765 binding sites were identified in cellular genomes in C666-1 cells, while 1,475 binding sites were characterized in cellular genomes in SNU719 cells. In all, 925 conservative binding sites were enriched from overlap analysis. (**D**) Overlap of EBNA1 ChIP-seq and RNA-seq in EBV^+^ epithelial cancer cells. Annotation of 925 consensus binding sites was achieved using the ChIPseeker package in the R program, which were localized in promoter, gene body, or distal intergenic regions of 837 cellular genes. Overlap analysis of EBNA1 ChIP-seq and RNA-seq characterized EBNA1-binding sites in promoter, intron, and distal intergenic regions of 14 differentially expressed genes. Motif analysis with HOMER identified a consensus palindromic-like sequence (GGCAGCATATGCTGCC, *P* < 0.01) in the center of EBNA1-binding sites. (**E**) ChIP-seq tracks for EBNA1-binding sites in CDC7 promoter, POU2F1 intron, and IL6R gene for Mutu1, C666-1, and SNU719 cells.

To investigate direct targets of VK1727 in 88 overlaps of differentially expressed genes, we re-analyzed previously published EBNA1 ChIP-seq data sets in MutuI, SNU719, and C666-1 cells (23, 24, 30). As expected, ChIP-seq analysis identified EBNA1-binding sites in FR, DS, and Qp regions in EBV genomes in three cell lines (Fig. 2B). We next identified EBNA1 ChIP-seq peaks that were observed in both C666-1 and SNU719 cells. Using bedtools, we identified 925 ChIP-seq peaks that were common to both C666-1 and SNU719 cells (Fig. 2C; Data File S2). ChIPSeeker-based annotation analysis identified consensus EBNA1-binding sites in promoter, gene body, and distal intergenic regions of 837 genes using a distance of <30 kb from the TSS as a window (Fig. 2D; Data File S2). After that, we performed integrated analysis of EBNA1 ChIP-seq and VK1727 RNA-seq data sets to identify differentially regulated genes with associated EBNA1 ChIP-seq peaks. EBNA1-binding sites were identified in promoter, intron, and distal intergenic regions of 14 differentially expressed genes in 88 overlaps across three VK1727 treatments, which were characterized as direct targets of VK1727 (Fig. 2D; Table S2). A conserved palindrome-like sequence (**GG**C**AG**CATATG**CT**G**CC**) was found in the center of EBNA1-binding sites with high confidence (*P* < 0.001; Fig. 2D; Table S2).

CDC7 and POU2F1 were recognized as the top two direct targets of EBNA1 across VK1727-treated PDX C15, C666-1, and SNU719 (Fig. 2D; Table S2). We, therefore, focused on these two target genes in more detail. EBNA1-binding sites were identified in the CDC7 promoter and the POU2F1 intron (Fig. 2E; Table S2). CDC7 is known as a key regulator for the initiation of DNA replication and the promotion of G1/S or G2/M transition (41). CDC7 is centrally located in each of the regulatory networks perturbed by VK1727 (Fig. S1C, S2C and S3C). POU2F1 (also known as OCT1) is described as a stem cell transcription factor and functionalizes in self-renewal in the context of colorectal cancer (42). IL6R, which was previously identified as an EBNA1-bound and regulated gene in EBV lymphoid cancer cell lines (24), was also found enriched in these EBV epithelial cancer cells (Fig. 2E; Table S2).

While EBNA1 can regulate some genes across multiple cell and tumor types, many of the genes were cell type specific. EBNA1-binding sites were observed to localize in the promoter, gene body, and distal intergenic regions of 69 upregulated and 28 downregulated (Fig. S6; Table S3) genes exclusive to SNU719 cells. In all, 25 EBNA1-bound targets were identified specifically for C666-1 cells (Fig. S7; Table S4). Eleven EBNA1-bound targets were identified exclusive to C15 cells (Fig. S8; Table S5). These findings suggest that EBNA1 engages both shared and cell-type-specific genomic targets across EBV-associated epithelial cancers.

### ChIP-qPCR validation of VK1727 reduced EBNA1 binding to CDC7 and POU2F1 genes

**Fig 3:**
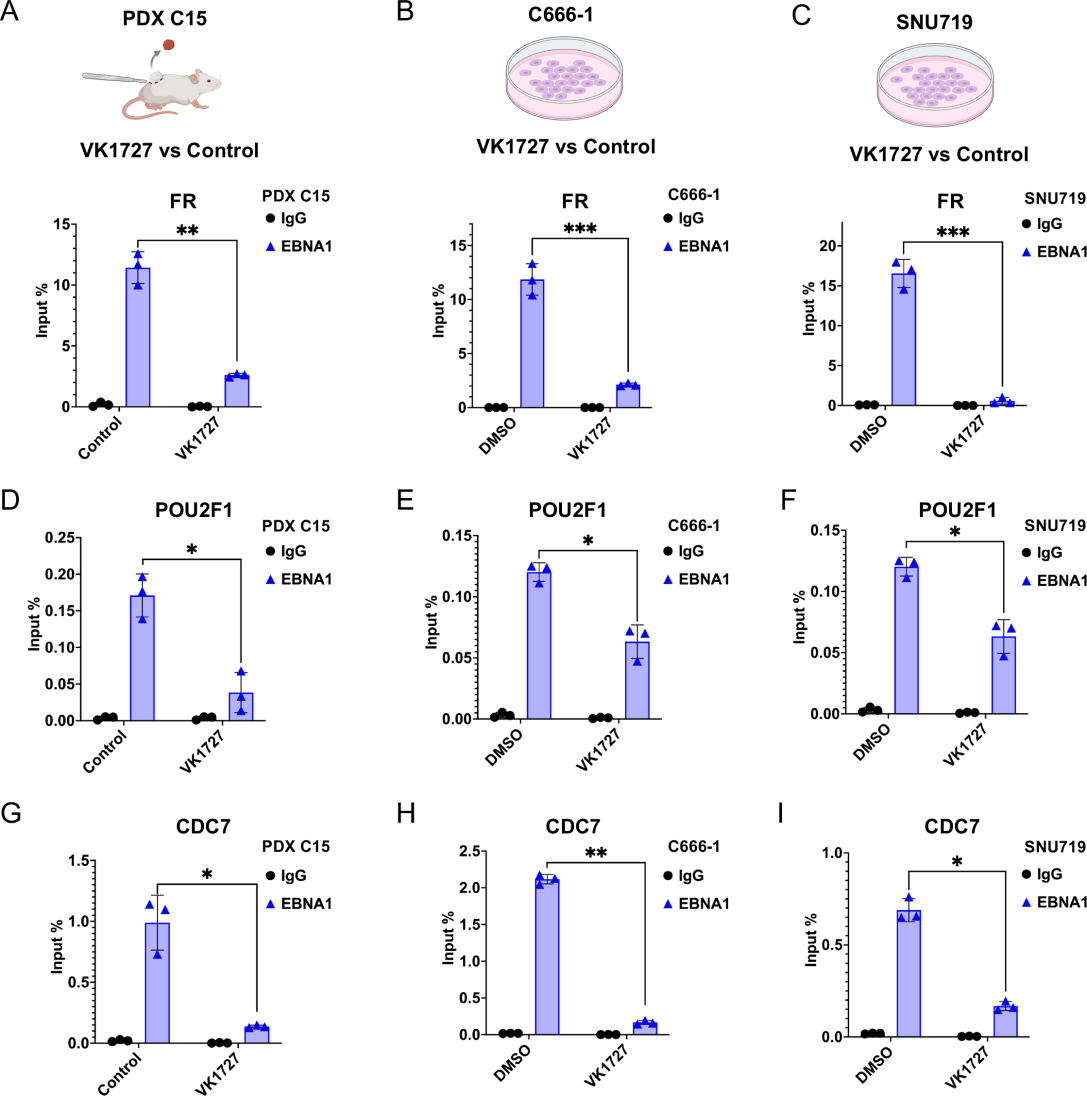
ChIP-qPCR validation of VK1727 reduced EBNA1 binding to the CDC7 promoter and POU2F1 intron. (**A–I**) ChIP-qPCR of EBNA1 or IgG binding to the EBV FR region (**A–C**), POU2F1 intronic region (**D–F**), or CDC7 promoter (**G–I**) in PDX C15 (**A, D, and G**), or C666-1 (**B, E, and H**), or SNU719 (**C, F, and I**). Error bars represent mean + SEM. Statistical comparisons between means were performed by Student’s *t*-test (two-tailed). *: *P* value < 0.05, **: *P* value < 0.01, ***: *P* value < 0.005, ****: *P* value < 0.001. *Y*-axis numerical scale labels denote ChIP-qPCR yield % input.

We next conducted ChIP-quantitative PCR (qPCR) assays to confirm VK1727 reduced EBNA1 binding to target regions. ChIP-qPCR assay showed VK1727 reduced over 70% of EBNA1 binding to the EBV FR region in PDX 15, C666-1, and SNU719 cells (Fig. 3A through C), and more than 60% of EBNA1 binding at the EBV DS and Qp regions in three cell models (Fig. S9A through F). The oriLyt region served as a negative control showing only background levels of EBNA1 binding at this site (Fig. S9G through I). These findings confirmed VK1727 efficiently reduced EBNA1 binding to known binding sites in the EBV genome. Next, we employed ChIP-qPCR to assay EBNA1 binding to the CDC7 promoter and POU2F1 intron in VK1727-treated PDX C15, C666-1, and SNU719 cells. We found that VK1727 reduced over 40% of EBNA1 binding to the POU2F1 intron (Fig. 3D through F) and ~80% of EBNA1 binding to the CDC7 promoter in three cell models (Fig. 3G through I).

### RT-qPCR and Western blot validation of VK1727 reduction of CDC7 and POU2F1 expression

**Fig 4:**
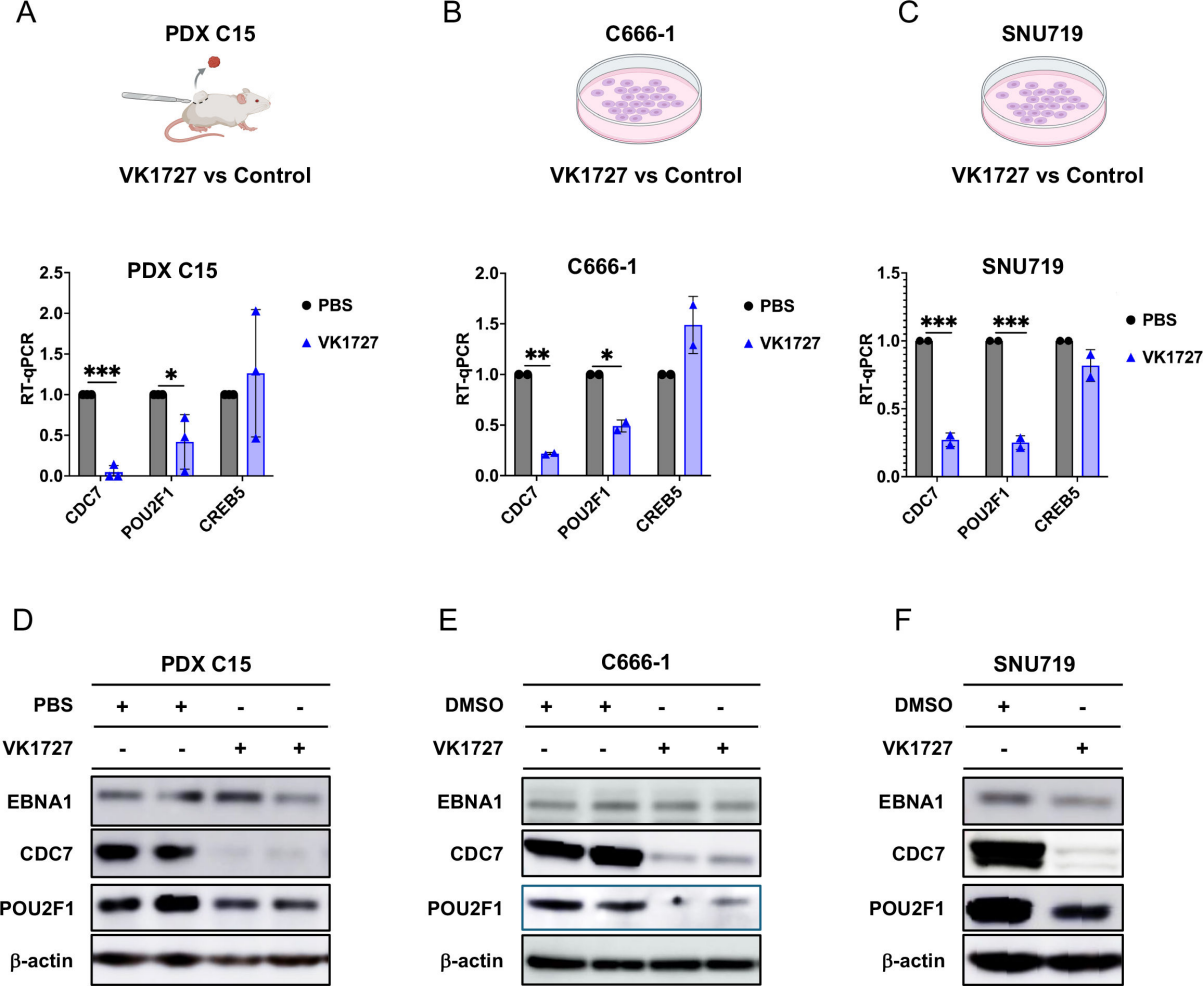
RT-qPCR and Western blot analysis of CDC7 and POU2F1 after VK1727 treatment. (**A–C**) RT-qPCR assay detected transcription of CDC7 and POU2F1 in PDX C15 after 5-day VK1727 treatments (**A**), C666-1 cells after 48-hour VK1727 treatments (**B**), and SNU719 cells after 48-hour VK1727 treatments (**C**). CREB5 was used as a control since it is a highly abundant transcript that is not affected by VK1727 treatment in these cell models, and lacks any EBNA1-binding sites. (**D–F**) Western blot probed for EBNA1, CDC7, POU2F1, and β-actin for PDX C15 (**D**), C666-1 cells (**E**), and SNU719 cells (**F**). β-Actin was used as a loading control in a Western blot. Error bars represent mean + SEM. Statistical comparisons between means were performed by Student’s *t*-test (two-tailed). *n* = 2 (biological duplicates with technical triplicates). *: *P* value < 0.05, **: *P* value < 0.01, ***: *P* value < 0.005.

To validate the RNA-seq data for VK1727 effects on CDC7 and POU2F1, we employed RT-qPCR assay and Western blot with VK1727-treated PDX C15, C666-1, and SNU719 cells (Fig. 4). RT-qPCR assay revealed a greater than 60% reduction of CDC7 transcription and ~50% reduction of POU2F1 transcription after VK1727 treatments (Fig. 4A through C). Western blot further confirmed a reduction of CDC7 and POU2F1 expression in VK1727-treated PDX C15, C666-1, and SNU719 cells (Fig. 4D through F), which is consistent with transcriptomic analysis above. EBNA1 protein levels were not significantly altered at these relatively early timepoints, suggesting that VK1727 does not destabilize EBNA1 protein and that the observed effects on CDC7 and POU2F1 are likely direct, rather than an indirect consequence of loss of EBV episomes or altered expression of other viral genes. In addition, VK1727 treatments did not reduce transcription levels of CDC7 and POU2F1 in EBV-negative NPC HK-1 cells (Fig. S10), demonstrating that the effects of VK1727 treatment are specific to EBV+ cells.

### EBNA1 domains required for transcription regulation of CDC7 and POU2F1

**Fig 5:**
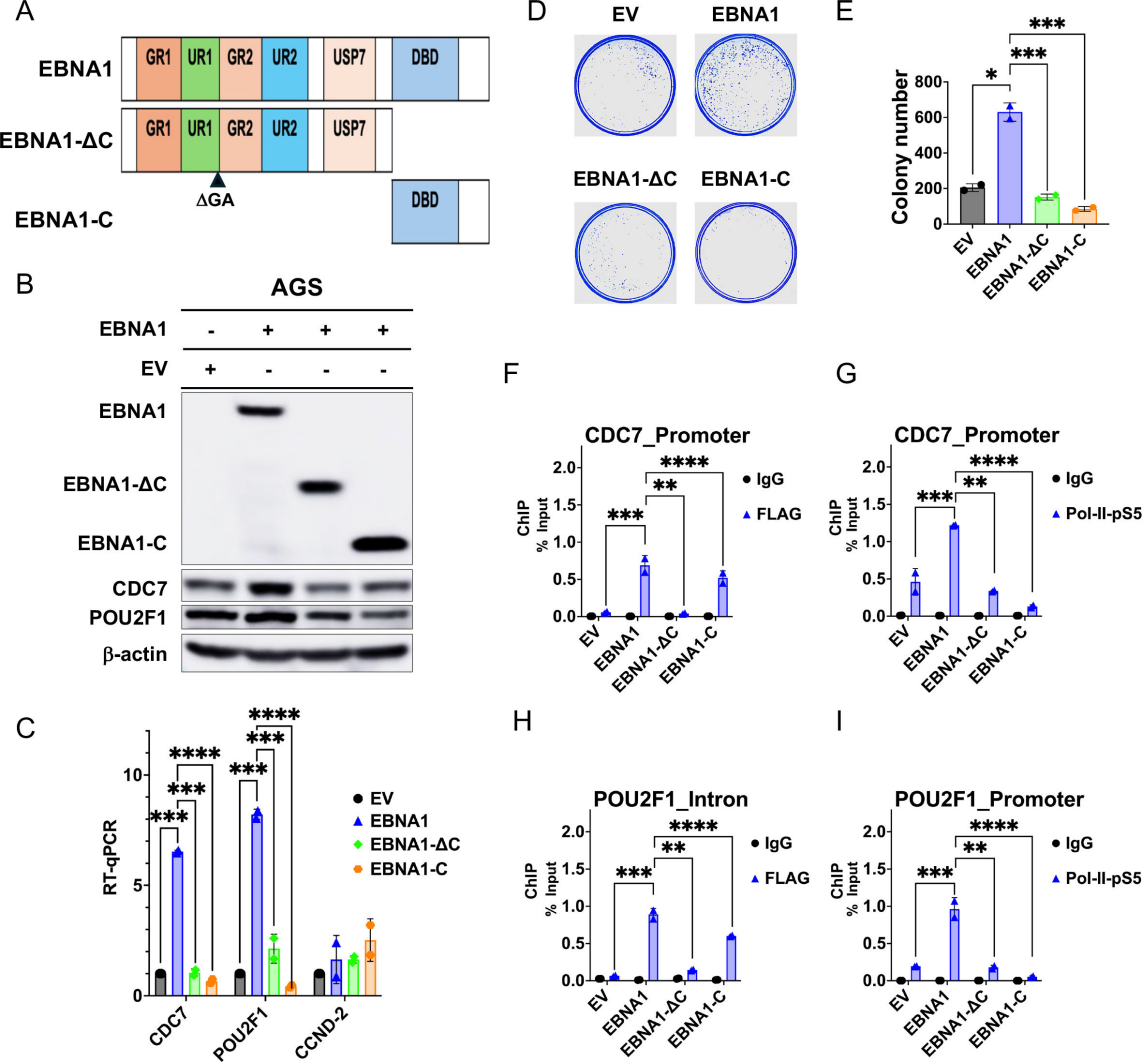
Structure-function analysis of EBNA1 regulation of CDC7 and POU2F1. (**A**) Schematic of EBNA1 domains and deletion mutants for FLAG-EBNA1, EBNA1-ΔC (aa1–459), and EBNA1-C (aa 460–641) for ectopic expression in AGS. (**B**) Western blot analysis of AGS cells transfected with pCMV-FLAG empty vector (EV) or FLAG-EBNA1, EBNA1-ΔC, or EBNA1-C and probed for FLAG (top panel), CDC7, POU2F1, or β-actin. Transfected AGS cells were collected for western blot analysis after 48-hour hygromycin B selection. (**C**) RT-qPCR assay measuring RNA for CDC7, POU2F1, and control cyclin D2 (CCND-2) gene in transduced AGS cells. (**D and E**) Colony formation assay detected proliferation of AGS cells transfected as in panels A and B. Quantification of colony forming assays is shown in panel E. (**F**) FLAG ChIP-qPCR assay for FLAG-EBNA1 binding to CDC7 promoter in transfected AGS cells. (**G**) RNA PoI II-pS5 ChIP-qPCR assay at the CDC7 promoter in transfected AGS cells. (**H**) FLAG ChIP-qPCR assay for FLAG-EBNA1 binding to POU2F1 intron in transfected AGS cells. (**I**) RNA Pol II-pS5 ChIP-qPCR assay at POU2F1 promoter in transfected AGS cells. IgG control was used for each of the ChIP-qPCR assays. Error bars represent mean + SEM. Statistical comparisons between means were performed by Student’s *t*-test (two-tailed). *: *P* value < 0.05, **: *P* value < 0.01, ***: *P* value < 0.005, ****: *P* value < 0.001. *Y*-axis numerical scale labels “% Input” denote ChIP-qPCR yield % input.

To gain insight into the mechanism by which EBNA1 regulates the transcription of CDC7 and POU2F1, we investigated the domain requirements of EBNA1. FLAG-tagged EBNA1 constructs, including full length lacking internal gly-ala repeats (WTΔGA), C-terminus deletion ΔC (lacking DBD), and C-terminus (DBD only), were overexpressed in EBV-negative AGS cells, with an empty vector (EV) as a control. All EBNA1 proteins were expressed at comparable levels, as confirmed by FLAG-Western blot (Fig. 5B). Western blotting further demonstrated that CDC7 and POU2F1 protein levels were upregulated in AGS cells transfected with EBNA1-WTΔGA, but not in cells expressing either deletion mutant (Fig. 5B). Consistent with this finding, RT-qPCR analysis showed significant upregulation of CDC7 and POU2F1 transcripts by full-length EBNA1, but not by the deletion mutants (ΔC or C-terminus) (Fig. 5C). EBNA1 had no detectable effect on CCND-2 (Cyclin D2) gene, indicating the effects are specific for the EBNA1-bound genes CDC7 and POU2F1 (Fig. 5C). We also assayed effects of EBNA1-WTΔGA and C-terminus deletion mutant (EBNA1-ΔC) in HK-1 cells (EBV-negative NPC cells), which also showed upregulation of CDC7 and POU2F1 by EBNA1-WTΔGA, but not by deletion mutant EBNA1-ΔC (Fig. S10B).

We next examined the functional consequences of EBNA1 on cellular proliferation using colony formation assays (Fig. 5D and E). Expression of EBNA1-WTΔGA promoted colony expansion of AGS cells compared with empty vector, while overexpression of mutant EBNA1-ΔC and EBNA1-C reduced AGS cell proliferation relative to empty vector (Fig. 5D and E). To further investigate the mechanism of transcription regulation, we performed FLAG and RNA polymerase II phosphorylated at Ser5 (RNA Pol II-pS5) ChIP q-PCR analysis. RNA Pol II-pS5 is a critical marker of the transition from transcription initiation to elongation (43). FLAG ChIP-qPCR demonstrated EBNA1-WTΔGA and EBNA1-C binding to CDC7 promoter and POU2F1 intron in transduced AGS cells, whereas EBNA1-ΔC showed no significant binding to these two target regions (Fig. 5F and H). In contrast, RNA Pol II-pS5 ChIP q-PCR revealed that EBNA1-WTΔGA enhanced RNA Pol II-pS5 recruitment to CDC7 and POU2F1 promoters in AGS cells compared with empty vector, while neither deletion mutant stimulated RNA Pol II-pS5 binding (Fig. 5G and I). Together, these findings demonstrate that the EBNA1 DNA-binding domain and N-terminal domains are both required for transcriptional activation of cellular target genes, and that recruitment of RNA Pol II-pS5 is a key component of this regulatory mechanism.

### Knockdown of POU2F1 impairs the function of EBNA1 in EBV-associated epithelial cancers

**Fig 6:**
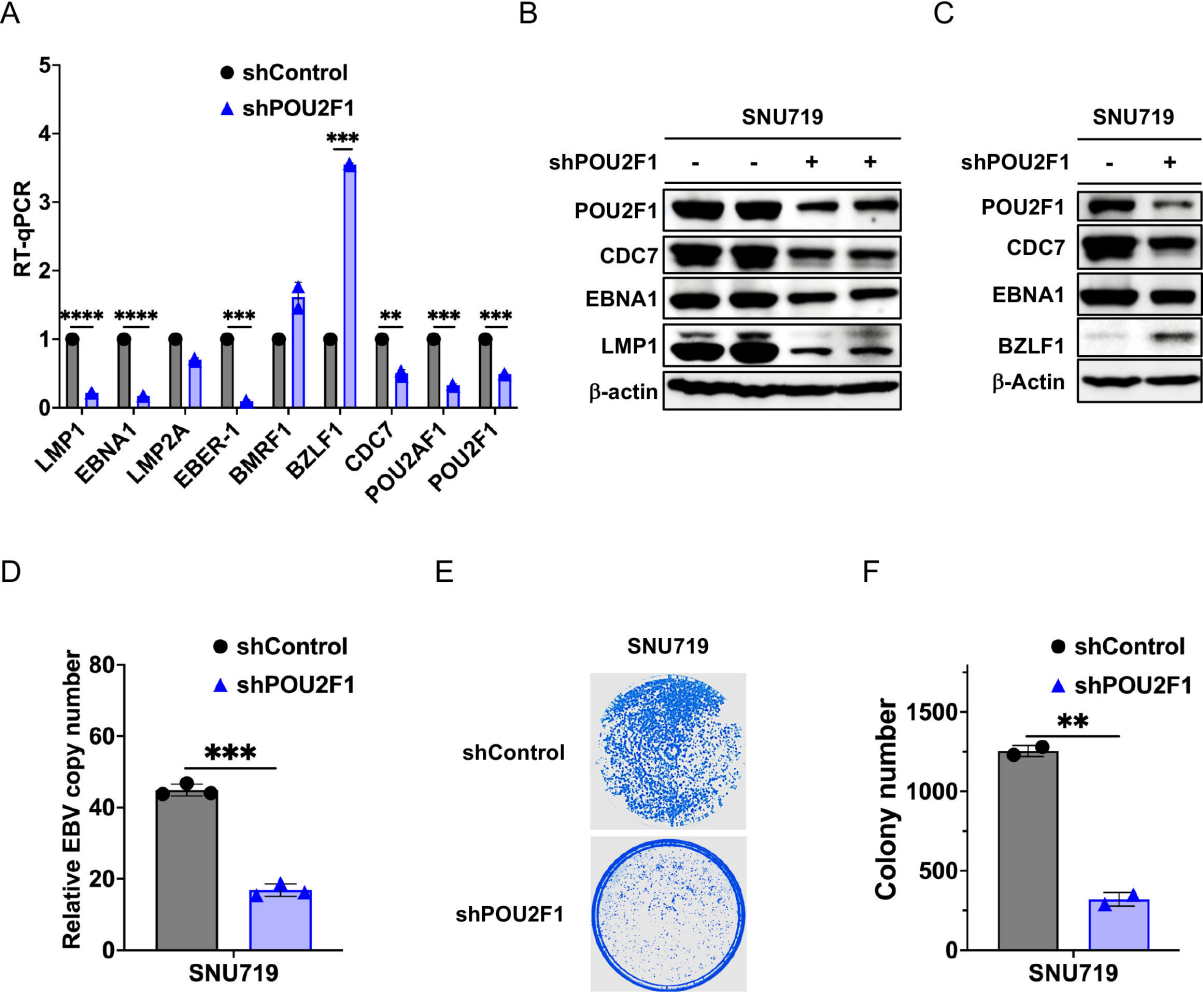
POU2F1 knockdown reduces EBV gene expression and colony growth formation in SNU719 cells. (**A**) RT-qPCR analysis of transcription of EBV genes (LMP1, EBNA1, LMP2A, EBER-1, BMRF1, and BZLF1) and cellular genes for CDC7, Pou2AF, and POU2F1 in SNU719 cells after knockdown with shPOU2F1 or shControl. (**B**) Western blot analysis of POU2F1, CDC7, EBNA1, LMP1, and β-Actin in SNU719 cells after knockdown with shPOU2F1 or shControl. (**C**) Western blot detects the expression of POU2F1, CDC7, EBNA1, CDC7, and BZLF1 in POU2F1 knockdown SNU719 cells. (**D**) Relative DNA qPCR assay measuring EBV DNA copy number in SNU719 cells after knockdown with shPOU2F1 or shControl. (**E and F**) Colony formation assay of SNU719 cells after knockdown with shPOU2F1 or shControl. Quantitation of colony formation shown in panel E. Error bars represent mean + SEM. Statistical comparisons between means were performed by Student’s *t*-test (two-tailed). *: *P* value < 0.05, **: *P* value < 0.01, ***: *P* value < 0.005, ****: *P* value < 0.001.

POU2F1 was identified as a direct target of EBNA1 gene regulation in three different EBV epithelial cancer cell models. To investigate the function of POU2F1 in EBV epithelial cancers, we employed small hairpin RNA (shRNA)–mediated knockdown of POU2F1 in SNU719 cells. A pool of three shRNA achieved approximately 50% knockdown of POU2F1 expression (Fig. 6A). RT-qPCR analysis revealed that knockdown of POU2F1 resulted in significantly reduced transcription of EBV genes (EBNA1, LMP1, EBER1, and EBER2) and cellular gene CDC7 in SNU719 cells (Fig. 6A), while activated transcription of BZLF1 and BMRF1 (Fig. 6A). Western blot analysis further confirmed that knockdown of POU2F1 resulted in a reduction of LMP1, EBNA1, and CDC7 protein levels (Fig. 6B), while activating expression of BZLF1 (Fig. 6C). We next assayed whether POU2F1 influences EBV genome replication or maintenance by measuring EBV DNA copy number using quantitative PCR and found that POU2F1 knockdown resulted in reduced copy number of EBV in SNU719 cells (Fig. 6D). These findings suggest that loss of POU2F1 can deregulate EBV latent and lytic transcription, but leads to a loss of viral copy number in SNU719 cells.

To investigate any potential effect of POU2F1 on EBNA1 DNA binding, we performed ChIP-qPCR to assay EBNA1 binding at EBV and cellular (e.g., CDC7) binding sites (Fig. S11). We found that knockdown of POU2F1 reduced EBNA1 binding at FR, DS, and Qp, as well as at the cellular binding site in CDC7 (Fig. S11). Previous studies have shown that OCT2 (POU2F2) contributes to EBNA1 binding and function at oriP in B-cells (44). Since POU2F2 may substitute for POU2F1 in B cells, we assayed the expression levels of POU2F1 and POU2F2 from RNA-seq data sets for each of our cell types (Fig. S12). As expected, POU2F1 mRNA is more abundant in EBV epithelial cells, while POU2F2 is more abundant in B cells. These findings suggest that POU2F1 can regulate EBNA1 DNA-binding in EBV epithelial cancer cells.

Since POU2F1 was described to maintain cancer stem cell (CSC) property in the context of colorectal cancer (42), we assayed colony formation of SNU719 (Fig. 6E and F) and 3D spheroid formation of C666-1 in soft agar (Fig. S13). Knockdown of POU2F1 significantly impaired SNU719 colony formation in SNU719 (Fig. 6E and F) and 3D spheroid formation for C666-1 (Fig. S13A). Treatment with VK1727 also reduced 3D spheroid formation in C666-1 (Fig. S13B). These results show that knockdown of POU2F1 impairs multiple functions of EBNA1 in maintaining EBV persistence and supports a multifactorial role of POU2F1 in EBV+ epithelial cancers.

### CDC7 inhibitors block cell proliferation in EBV+ epithelial cancer cell lines

**Fig 7:**
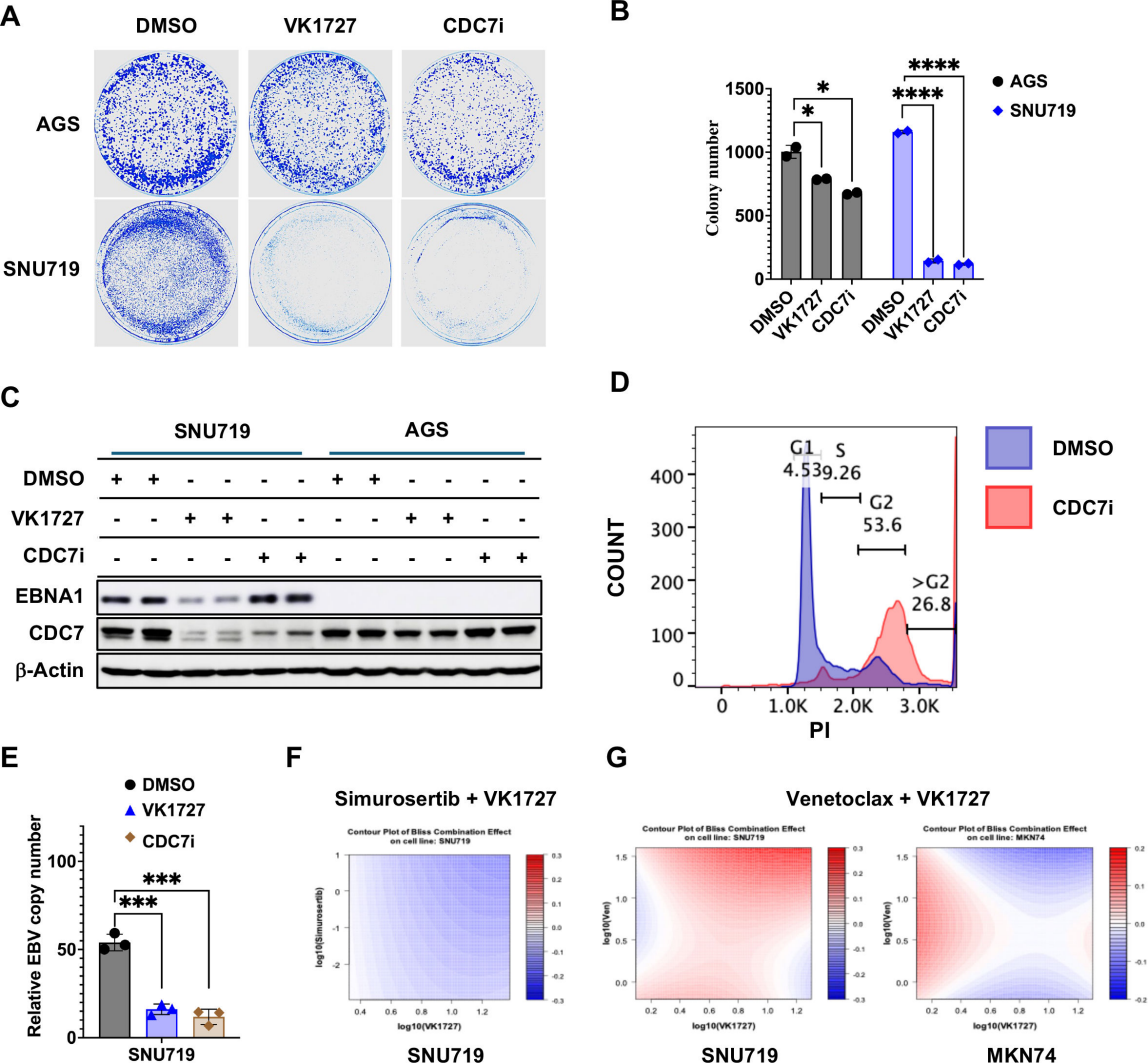
EBNA1 inhibitors phenocopy the CDC7 inhibitor simurosertib and are synergistic with the Bcl2 inhibitor venetoclax. (**A and B**) Colony formation of SNU719 cells after treatment with VK1727 (20 μM) or CDC7 inhibitor simurosertib (1.5 μM). Error bars represent mean + SEM. Statistical comparisons between means were performed by Student’s *t*-test (two-tailed). *: *P* value < 0.05, ****: *P* value < 0.001. (**C**) Western blot analysis of EBNA1 and CDC7 in VK1727 or CDC7 inhibitor–treated AGS and SNU719 cells. Simurosertib (CDC7i) treatment was 1.5 μM for 48 hours, VK1727 treatment was 20 μM for 48 hours. (**D**) Flow cytometry analysis revealed G2/M arrest in SNU719 cells after CDC7i treatments. SNU719 cells were treated with 1.5 μM CDC7i for 48 hours. Propidium Iodide (PI) was employed to stain genomic DNA in SNU719 cells. (**E**) Relative DNA-qPCR assay to measure the copy number of EBV in SNU719 cells. Error bars represent mean + SEM. Statistical comparisons between means were performed by Student’s *t*-test (two-tailed). ***: *P* value < 0.005. (**F and G**) Bliss synergy analysis of combination treatment with VK1727 and simurosertib on SNU719 cell growth (**F**) or VK1727 and venetoclax on SNU719 (left) and MKN74 (right) cell growth. Resazurin assay was used to measure cell viability under different combination treatments of VK1727 and venetoclax. Bliss synergy score was calculated using the SynergyFinder package (3.0) in the R program.

To address the potential role of CDC7 in EBV^+^ epithelial cancers, we used a pharmacological approach with the CDC7 inhibitor (CDC7i) simurosertib (45). We first used a resazurin-based cell metabolism assay to show that 1.5 µM simurosertib selectively inhibited EBV^+^ SNU719 and C666-1 relative to EBV^−^ paired cell lines AGS and HK-1, respectively (Fig. S14A and B). We then used a colony formation assay to show that 1.5 µM simurosertib significantly inhibited colony expansion in SNU719 cells, while only modestly affecting colony growth in AGS cells (Fig. 7A and B). Similar selective effects were observed in C666-1 (EBV^+^) compared to HK-1 (EBV^−^) cells (Fig. S14C and D). This selectivity was also observed with 20 µM VK1727. Western blot analysis revealed that VK1727 and simurosertib (CDC7i) significantly (~4-fold) reduced CDC7 expression in SNU719 cells at 72 hours (Fig. 7C), but had only modest (<1.5-fold) effects on CDC7 in AGS cells. VK1727 also reduced EBNA1 expression in SNU719, but this was not observed with simurosertib (Fig. 7C). Furthermore, we observed two bands of CDC7 in SNU719 cells rather than AGS cells (Fig. 7C), and simurosertib significantly inhibited the expression of the lower band (Fig. 7C). This suggests that simurosertib, but not EBNA1, inhibits autophosphorylation of CDC7 in EBV^+^ gastric cancers. Flow cytometry identified a G2/M cell cycle arrest in simurosertib-treated SNU719 (Fig. 7D). In some cells, simurosertib appeared to impair chromosome segregation, resulting in an accumulation of cells with 2N DNA content (Fig. 7D). We next measured EBV DNA copy number by DNA qPCR assay and observed a reduction of viral genome copy number in both VK1727 and simurosertib-treated SNU719 cells (Fig. 7E).

To determine whether simurosertib and VK1727 act in the same pathway, we performed a resazurin assay to detect the combination of different concentrations of simurosertib and VK1727 for the inhibition of the growth of SNU719 cells and assessed their combined effects using a two-stage response surface model. This tests the impact of different combinations of various concentrations of the two drugs on cell viability. The combination of simurosertib and VK1727 did not exhibit synergistic growth inhibition in SNU719 cells (Fig. 7F), which is consistent with both agents acting on overlapping pathways. In contrast, CDC7 inhibitors have been reported to act synergistically with BCL2 inhibitors (e.g., venetoclax) in acute myeloid leukemia (AML) (46). We therefore tested whether VK1727 would similarly synergize with the Bcl2 inhibitor, venetoclax. We detected synergistic effects of combination with different concentrations of VK1727 (0–40 µM) and venetoclax (0–40 µM) on the inhibition of the growth of SNU719 cells. 20 µM VK1727 exhibited strong synergistic activity with venetoclax (20–40 µM) on the inhibition of the growth of EBV+ SNU719 cell lines (Fig. 7G, left), whereas no such synergy was observed in EBV-negative MKN74 cells, suggesting that this effect is EBV dependent (Fig. 7G, right). Together, these findings suggest that simurosertib and VK1727 inhibit the same pathways controlling cell cycle progression and viral DNA replication while venetoclax and VK1727 inhibit complementary and therefore synergistic pathways in EBV+ epithelial cancers.

### CDC7 and POU2F1 are EBNA1-regulated genes in EBV+ B-lymphoma cell line MutuI

To determine whether EBNA1 regulation of CDC7 and POU2F1 in epithelial cancers is conserved in B-cell lymphoma, we analyzed RNA-seq from MutuI cells treated with the EBNA1 inhibitor VK1850. VK1850 is a close analog of VK1727 and is expected to function through an identical mechanism by binding EBNA1 and blocking its DNA binding. Comparative analysis across 4 treatment conditions identified 6 consistently upregulated genes (Fig. S15A) and 15 consistently downregulated genes (Fig. S15B). Pathway analysis of these 21 differentially expressed genes identified common signaling pathways (*P* < 0.05), and the top four signaling pathways included cell cycle, DNA replication, pyrimidine metabolism, and the P53 signaling pathway (Fig. S15C). Western blotting confirmed that EBNA1 inhibition significantly reduced the expression of CDC7 and POU2F1 in MutuI cells following 72 hours of treatment with VK1727 (Fig. S15D). Together, these findings indicate that cell cycle pathways represent conserved targets of EBNA1 and CDC7 that are selectively downregulated upon EBNA1 inhibition in both EBV+ epithelial and B-cell lymphoma contexts.

## DISCUSSION

EBV is a well-established human tumor virus due to its association with several human cancers, but its mechanisms of oncogenesis, particularly in epithelial cancers, are not fully understood (47). In EBV epithelial cancers, EBV episomes and EBNA1 protein are present in virtually all cancer cells (48, 49). EBNA1 is thought to make essential contributions to the oncogenic process by maintaining the viral episome and by binding cellular genomic sites to regulate host genes that are critical for oncogenic transformation (18, 50). In this study, we leveraged EBNA1 inhibitors as pharmacological tools to investigate the functional targets of EBNA1 in three different models of EBV-associated epithelial cancers. VK1727 and its closely related analog VK2019 target the DNA-binding domain of EBNA1 (37, 38). Our findings further validate VK1727 as an inhibitor of EBNA1 DNA binding in both cell culture and PDX tumor models of nasopharyngeal carcinoma (e.g., PDX C15).

We analyzed the transcriptomic response to VK1727 in C666-1, SNU719, and PDX C15 and identified common genes and pathways that were differentially regulated by drug treatment. Patient-derived xenograft C15 is a transplantable EBV^+^ NPC tumor model that is sustained in NOD mice (NOD.*Cg-Prkdc^scid^ Il2rgtm1Wjl/ScJ*) for tumor growth and sustains a high copy of EBV episomes (36). C666-1 cell line is an NOD mice-passaged NPC tumor-derived cell line and stably maintains EBV persistence *in vitro* (39). SNU719 cell line is derived from EBV-associated gastric carcinoma and consistently maintains EBV persistence (40). All three EBV+ epithelial cancer models shared a common response to EBNA1 inhibitor VK1727, with cell cycle regulation, cellular senescence, and transcriptional dysregulation in cancer, p53 signaling, and EBV virus infection scoring among the most significant. By integrating EBNA1 ChIP-seq data with known EBNA1-binding sites, we identified a small number of candidate genes with a high probability of being directly bound and transcriptionally regulated by EBNA1. We identified CDC7 and POU2F1 as among the top candidates and demonstrated that EBNA1 binds and regulates these genes in all three cell models. Moreover, both genes make essential contributions to EBV+ epithelial cancer cell growth and survival.

Previous studies have identified EBNA1-bound transcriptional targets in lymphoid models of EBV cancer (24), including IL-6R, MEF2B, and EBF1, each of which plays an important role in B-cell development and lymphomagenesis. In the present study, IL-6R was observed significantly downregulated in EBV+ epithelial cancers (Table S2); however, other studies suggest that IL-6R plays a more significant role in immune cells (51). Neither MEF2D nor EBF1 scored as highly significant in epithelial cancers, suggesting that the action of EBNA1 is host cell or tumor type dependent. Consistent with this idea, many EBNA1-regulated genes in NPC and EBVaGC were not shared between models and likely reflect cell-type-specific regulation. EBNA1 DNA binding to host genes also varied across cell types, which may further contribute to differences in gene regulation. Many differentially regulated genes did not contain EBNA1-binding sites, likely reflecting indirect effects of EBNA1 inhibition on upstream transcriptional regulators, as well as peaks below threshold detection. In addition, many EBNA1-binding sites were located in intergenic regions that could not be assigned to any specific gene or distal enhancers, while some EBNA1-bound promoter sites did not correlate with transcriptional changes. Consequently, the overlap between EBNA1 binding and transcriptional deregulation is relatively limited. While this may suggest that EBNA1 is not a strong transcriptional regulator compared to EBNA2 or EBNA3C, which are known to form super-enhancers at many EBV-regulated genes in B-lymphocytes (52, 53), we nonetheless identified a distinct subset of EBNA1-bound genes that are differentially regulated (Tables S3 to S5). Here, we focused on the genes that are commonly regulated in the three epithelial cancer models to test the hypothesis that EBNA1 controls a set of essential, conserved targets in epithelial oncogenesis.

Our integrated data identified the cell cycle-dependent kinase CDC7 and the stem cell transcription factor POU2F1 as direct targets of EBNA1-mediated gene regulation in all EBV epithelial cancer models examined (Fig. 2E, 3G through I, 4 and 8). EBNA1 binding at the CDC7 promoter or the first intron of POU2F1 enhanced RNA Pol II-pS5 binding at the transcription start sites of these genes (Fig. 5), suggesting that EBNA1 contributes to the transcriptional activation of these genes. CDC7 has emerged as an attractive therapeutic target in several cancers, including pancreatic, prostate, breast, and lung cancers (54–57). CDC7 has a well-established function in replication origin firing by phosphorylating components of the MCM complex required for licensing and initiating DNA replication (58, 59). The MCM complex is also known to associate with EBV oriP and promotes viral replication (60, 61). Our study suggests CDC7 as a promising therapeutic target for EBV+ epithelial cancers, since the CDC7 inhibitor simurosertib reduced colony expansion and blocked G2/M progression in EBV+ epithelial cells (Fig. 7; Fig. S14C and D). Together with previous studies, our findings support a model that CDC7-mediated phosphorylation of the MCM complex contributes to the maintenance and persistence of EBV, consistent with the observation that simurosertib phenocopies EBNA1 inhibition in reducing EBV DNA copy number (Fig. 7E).

EBNA1 was also found to bind and regulate POU2F1 in each of the epithelial cancer models tested here. POU2F1 (also known as OCT1) has been reported to promote cancer stem self-renewal properties and resistance to chemotherapy (62). Our results suggest the POU2F1 pathway could be a therapeutic vulnerability in EBV+ tumors, as POU2F1 knockdown reduced cell proliferation, viral copy number, and viral gene expression (Fig. 6). POU2F1 often cooperates with POU2F2 (known as OCT2) to stimulate transcription (63). OCT2 has been shown to bind to oriP in EBV genomes and regulate transcription of EBV genes in EBV+ lymphoid cancers and transformed B cells (44). We found that POU2F1 (OCT1) depletion led to a loss of EBNA1 DNA binding in ChIP assay (Fig. S11), suggesting that it may facilitate EBNA1 binding directly or indirectly. POU2F1 is expressed at higher levels than POU2F2 (OCT2) in EBV+ epithelial cancers (Fig. S12A), while POU2F2 is transcribed higher than POU2F1 in lymphoid cell line MutuI (Fig. S12D). VK1727 treatments reduced transcription of POU2F1 across EBV+ epithelial and B lymphoma cell lines (Fig. 4; Fig. S14 and S15) but stimulated transcription of POU2F2 in MutuI cells (Fig. S15). Thus, OCT1 and OCT2 may act cooperatively in B cells, whereas OCT1, alone or in combination with other OCT members, may be sufficient in EBV+ epithelial cancers to support EBNA1 DNA or chromatin binding, promote stem cell self-renewal, and viral oncogenesis (64). Notably, OCT1 has also been investigated as a target for cancer therapy, and early-stage small-molecule inhibitors have been developed for hepatocellular carcinomas (65).

Our study demonstrates that EBNA1-mediated transcriptional activation of CDC7 and POU2F1 correlates with increases in RNA Pol II-pS5 occupancy at transcription start sites (Fig. 5). Deletion of either the C-terminal DNA-binding domain or the N-terminal tethering domains resulted in a loss of transcription activation function (Fig. 5). Previous studies have identified key functions of the EBNA1 N-terminal region in chromosome tethering (66), transcriptional activation (67), nuclear localization (68), as well as interactions with histone H1 (69, 70), BRD4 (71), USP7 (72), CK2 (73, 74), and RNAs (75, 76), including G-quadruplexes (77, 78). Although we did not observe transcriptional changes associated with all EBNA1-binding sites, it remains possible that EBNA1 influences many genes through distal regulatory elements. Future studies will be needed to map long-range EBNA1 interactions and better define their contribution to transcriptional regulation.

**Fig 8:**
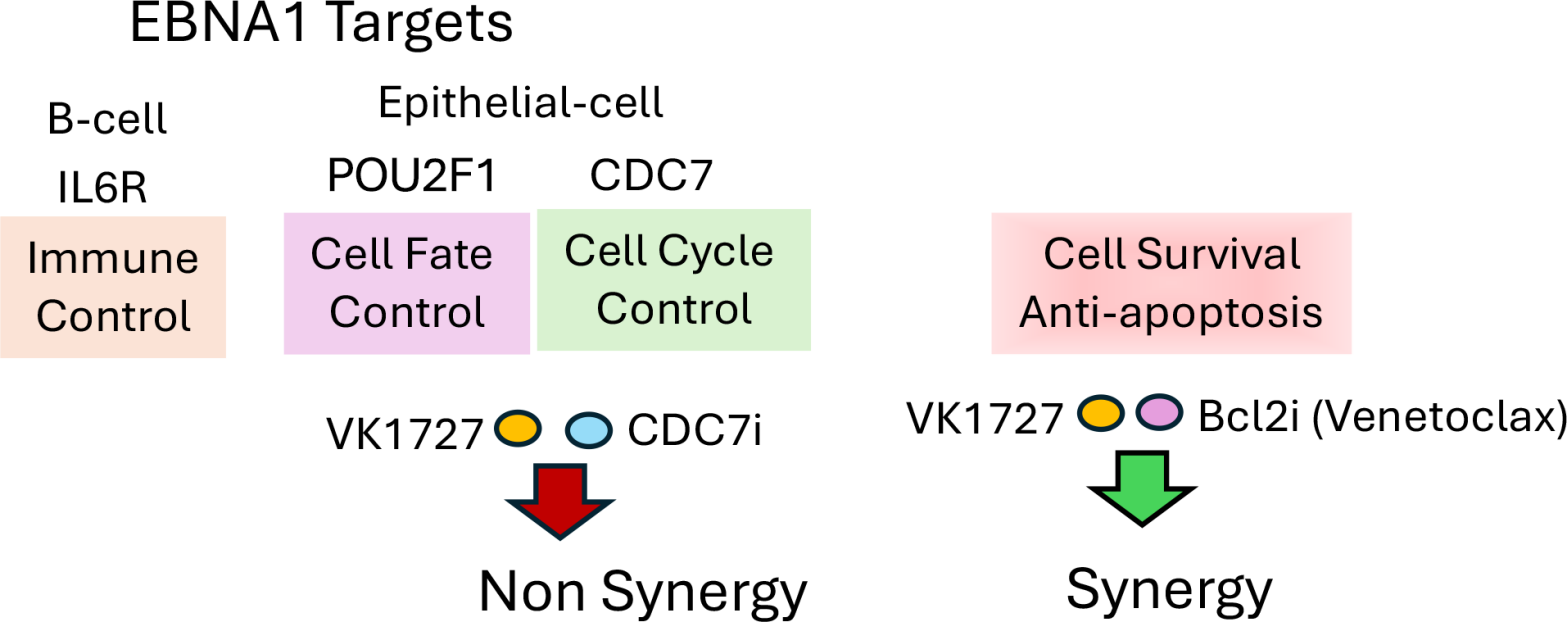
Model for EBNA1 directly bound cellular gene targets and drug synergy pathways. EBNA1 stimulates transcription of CDC7 to control cell cycle progression and activates the expression of POU2F1 to regulate cell fate in EBV^+^ epithelial cancers. CDC7 inhibitors are non-synergistic and phenocopy EBNA1 inhibitors in epithelial cells. EBNA1 inhibitors are synergistic with Bcl2 inhibitors, which are not direct targets of EBNA1. Previous studies found EBNA1 regulated immune control genes in B-lymphocytes.

In conclusion, the studies presented here further demonstrate that EBNA1 inhibitors, such as VK1727, effectively block EBNA1 binding and perturb host gene expression through both direct and indirect mechanisms. Importantly, we have identified a conserved set of EBNA1 target genes across multiple EBV+ epithelial cancer models that may serve as targets for therapeutic intervention. Some of these EBNA1 targets may also be activated in EBV-negative tumors through EBNA1-independent mechanisms. Our findings further suggest rational combination strategies, such as pairing EBNA1 inhibitors with Bcl2 inhibitors like venetoclax, which is also known to synergize with CDC7 inhibitors (46) (Fig. 7 and 8). EBNA1 inhibitors may also enhance the efficacy of CAR T-cell (79) and monoclonal antibody therapies (80) targeting additional cellular pathways identified in this study, offering new avenues for the treatment of EBV-associated epithelial cancers.

## MATERIALS AND METHODS

### Cell lines, patient-derived xenograft C15, and EBNA1 inhibitor VK1727 treatments

EBV latently infected cell lines (C666-1, EBV+ nasopharyngeal carcinoma cell line; SNU719, EBV+ gastric cancer cell line) and EBV-negative cell lines (HK-1, nasopharyngeal carcinoma cell line; AGS, gastric cancer cell line) were cultured with Gibco RPMI 1640 medium (Catalog number 11875101) containing 10% fetal bovine serum (FBS; Gibco, Gaithersburg, MD, USA), penicillin, streptomycin (50 U/mL), and L-glutamine at 37°C in 5% CO_2_ humidified incubators. PDX C15 was described previously (37), and transplanted into NOD mice (NOD.*Cg-Prkdc^scid^ Il2rgtm1Wjl/ScJ*) for sustaining tumor growth *in vivo* according to the previous description (36).

NOD mice (*n* = 3) with engrafted PDX C15 tumors were treated with either vehicle control or 10 µM VK1727 injected every 12 hours for a total of 5 days. C666-1 cells were treated with 10 µM VK1727 or DMSO control every 12 hours for a total of 2 days. SNU719 was treated with 20 µM VK1727 or DMSO control every 12 hours for a total of 2 days, as SNU719 cells maintain a higher copy number of EBV episomes than C666-1 cells. Triplicates were performed for each condition (VK1727 and DMSO).

Cells were treated with CDC7 inhibitor simurosertib (TAK-931; Catalog number, HY-100888) dose range 0.1– 10 μM or Bcl2 inhibitor venetoclax dose range of 0.6–40 μM to assess synergy with VK1727.

### RNA-seq, differentiation gene expression, and functional analysis

Total RNAs were extracted from PDX C15, SNU719, and C666-1 cells using QIAGEN RNeasy Mini Kit (Catalog number, 74104), which were treated with VK1727 and DMSO. One microgram total RNA of each sample was used for library construction using Illumina Stranded Total RNA Prep (Ligation with Ribo-Zero Plus) Kit (Catalog number, 20040529). Ribosomal RNA depletion, RNA fragmentation, 3′ and 5′ end repairing, reverse transcription into cDNA, and adaptor ligation were achieved according to provided protocols (support.illumina.com). An optimized PCR program (optimization of PCR cycles) was applied for amplification of cDNA libraries. The cDNA libraries were purified using AMPure XP Beads for DNA Cleanup (Catalog number, A63880) according to the manufacturer’s protocols (support.illumina.com). The quality of the cDNA library was measured using the 4200 TapeStation System (Agilent). High-Sensitivity D1000 DNA ScreenTape assay (Catalog number, NC1786959) was used to quantify the average size and concentration of the cDNA library. Pair-end sequencing was conducted to identify sequences of cDNA reads in the library by the Genome Center in the Wistar Institute.

The fastq files of RNA-seq raw data sets were obtained from the FTP database provided by the Genome Center in Wistar Institute. The quality of RNA-seq raw data sets was determined using FastQC (version, 1.0.0) coupled with MultiQC. Adaptor sequences from each read were removed using the Trim Galore (version 0.6.10) program with a pair-end trimming algorithm. RNA reads were mapped to integrated human (Hg19, also known as GRCh37) and EBV (NC007605) reference genomes using the STAR aligner (version 2.7.11b). The count table of reads per gene was generated using the featureCounts program (81) and utilized for subsequent analysis (e.g., differential gene expression and functional analysis). The DESeq2 package (version, 1.50.0), embodied in the R (version, 4.5.2) program, was used for differential gene expression analysis (82). The EnhancedVolcano and heatmap.2 packages were employed to generate volcano plots and heatmaps for visualization (83). The clusterProfiler package was applied to decode the biological function of differentially expressed genes in KEGG database (84). The DOSE package was utilized to generate bar plots and networks of enriched biological functions (85). BioVenn (86) was used to identify overlapping targets of downregulated and upregulated genes across VK1727-treated PDX C15, C666-1, and SNU719 cells.

Differential expression analysis was conducted using DESeq2 on raw count data, where genes with fewer than 10 counts were filtered out, and significance was defined by an adjusted *P*-value < 0.05 (82). To identify shared and unique gene sets, BioVenn (86) was employed to identify overlaps of upregulated and downregulated genes across four EBNA1 inhibitor treatments. The Enrichr database was used for KEGG pathway enrichment of overlaps of upregulated and downregulated genes (87).

### Integrated analysis between EBNA1 ChIP-seq and RNA-seq data sets

EBNA1 ChIP-seq fastq files of SNU719 and MutuI cells were downloaded from the FTP database provided by the Genome Center in the Wistar Institute, which was already published from our lab (GSE289711) (30). EBNA1 ChIP-Seq of C666-1 cells was consistent with the previous description (30). The fastq files of EBNA1 ChIP-seq raw data sets were retrieved from the FTP database provided by the Genome Center in the Wistar Institute. Quality control and trimming steps were conducted using FastQC and Trim Galore (version 0.6.10) programs. BowTie2 was employed to map DNA reads to the integrated human (Hg19) and EBV (NC007605) reference genomes using reported protocols (24). Samtools and Picard were used to remove duplicate reads in BAM files. The MACS2 program was utilized to call EBNA1 peaks in cellular (Hg19) and viral (NC007605) genomes (24). The ChIPSeeker package, embodied in the R program, was used to annotate EBNA1-binding sites in narrow peak files to cellular genomes and generate an annotation table for EBNA1-binding sites (88). Then integrated analyses were performed to identify EBNA1-binding sites in the promoter, gene body, and distal intergenic regions of upregulated or downregulated genes under VK1727 treatments, which employs the BioVenn program.

### ChIP assay for identifying EBNA1 and RNA polymerase II CTD phosphorylated Ser5 binding to viral and cellular genomes

C666-1 or SNU719 cells (10^6^) were seeded on six-well plates. Three wells of C666-1 or SNU719 cells were treated with 0.3% DMSO; the other three were dealt with 10 or 20 µM VK1727 every 12 hours for a total of 2 days. Treated C666-1 or SNU719 cells were collected for ChIP assay using Gibco RPMI 1640 medium (Catalog number 11875101). The ChIP assay was accomplished based on reported protocols from our lab (30). Q-PCR was used to measure EBNA1 binding to FR, DS, Qp, and OriPLytic in the viral genome using site-specific primers (30). Primers were designed to verify EBNA1 binding to the promoter region of CDC7 and the first intron region of POU2F1 in EBV epithelial cancers (Table S6). The Q-PCR running program was conducted in accordance with previous descriptions (30). Input percentage was utilized to determine differences in EBNA1 binding to viral and cellular genomes between VK1727 treatment and control groups. Input percentage was calculated using 2^−ΔCT^. The ΔCT was generated through differences in CT values between IP samples (IgG or EBNA1) and input samples. Moreover, the ChIP assay was employed to identify EBNA1 binding to genomic regions of interest in control and VK1727-treated C15 tumors.

ChIP-qPCR followed previously described protocols (89). Primers were designed to target regions close to TSS (from −1,000 bp to 1,000 bp) of CDC7 and POU2F1 (Table S6). The ChIP assay was conducted using an antibody against RNA Pol-II pS5 (Catalog number, 2687451; Active Motif). Input percentage was utilized to determine differences in RNA Pol-II PS5 access to the promoter of CDC7 and POU2F1 between control and treatments.

### Transient transfection of wild type, C-terminus deletion ΔC, and C-terminus of EBNA1 in AGS and HK-1 cells

Wild-type full length lacking internal gly-ala repeats (WTΔGA), C-terminus deletion ΔC (lacking DBD, deletion from 460 to C-terminus 641), and C-terminus (DBD only, deletion from N-terminus 2 to 459) of FLAG-EBNA1 with OriP have been described previously (90). The empty vector pCMV-FLAG (pPL748) was used as a control. The hygromycin B-resistant gene was cloned into the backbone of four plasmids. Four plasmids were transiently transduced into AGS and HK-1 cells using the Lipofectamine 2000 kit (Catalog number 11668027, Invitrogen). 200 µg/mL hygromycin B (Catalog number 10687010, Gibco) was used to select AGS cells and HK-1 with overexpression of wild type, ΔC, and the C-terminus of FLAG-EBNA1.

### POU2F1 knockdown and selective effects of CDC7 inhibitors on EBV+ epithelial cancers

shRNAs were employed to knock down POU2F1 in SNU719 cells. The shRNAs were purchased from the Wistar Institute collection of Sigma shRNA library (Table S6). Lentivirus production and purification were performed as described previously (91). Purified lentivirus, cloned with POU2F1 shRNAs or scramble control, was recruited to infect SNU719 cells for 48 hours, which were cultured with Gibco RPMI 1640 Medium (supplemented with 10 µg/mL polybrene, Catalog number K2701). 2 µg/mL puromycin (Catalog number A1113803, Gibco) selection was utilized to obtain the POU2F1 knockdown pool of SNU719 cells. RT-Q-PCR assay and western blot were conducted to quantify the expression of POU2F1 and other genes of interest in control and POU2F1 knockdown SNU719 cells.

### Resazurin assay and synergy analysis

Resazurin viability assay was performed as described previously (90). Briefly, 8,000 EBV+ and EBV− cancer cells were seeded into each well in a 96-well plate. Different concentrations of simurosertib (100 nM–10 μM) were used to treat EBV+ and EBV− cancer cells for over 9 days. Resazurin sodium salt (Catalog number, R7017-1G) was used to identify cell viability. The optical value of cell viability was obtained using the PerkinElmer EnVision Xcite multilabel plate reader. Puromycin dihydrochloride (Catalog number A1113803) was utilized as a positive control to normalize the inhibition of cell viability. The percentage of growth inhibition was calculated in accordance with a previous study (37).

Synergy analysis of VK1727 and simurosertib was performed using the resazurin viability assay. A combination of different concentrations of VK1727 and simurosertib was employed to inhibit the cell viability of SNU719 cells. The percentage of growth inhibition was calculated as described in a previous study (37). The SynergyFinder package (3.0) in the R program was utilized to analyze synergistic effects between two inhibitors using a two-stage response surface model (92).

### Colony formation and soft agar assay

For colony formation assays, 2,000–3,000 cells of EBV^+^ and EBV-negative epithelial cancer cells were seeded on 60 mm × 15 mm Corning tissue-culture-treated culture dishes (CLS430166-500EA) for 7 days. Then, 10 µM VK1727 and 1.5 µM (selective dose) simurosertib were prepared to treat cell clones on 50 × 15 mm Petri dishes every 12 hours for a total of 7 days. DMSO (0.3%) was used for the control group. Duplicates were conducted for each condition (VK1727 and simurosertib treatments or POU2F1 knockdown). After that, cell clones were fixed with 10% formaldehyde (SKU: C3966-1L) for 10 min. The fixed cell clones were washed with PBS and stained with 0.1% crystal violet (Catalog number, V5265-2; Sigma) for 10 min. The Biorad ChemiDoc MP Imaging System was used to scan cell clones on 50 × 15 mm petri dishes and generate TIFF files for quantitative analysis. The ImageJ program (version 1.54g) was used to determine the number of cell clones on 50 × 15 mm petri dishes. Student’s *t* test was used to identify numeric differences of cell clones between control and treatments using Prism-GraphPad (Version 10). *P* value was utilized to measure significance of differences between control and treatments.

Soft agar assays were performed to identify sphere formation of C666-1 cells in accordance with previous description (93). UltraPure low melting point agarose (Catalog number, 16520100) and Gibco RPMI 1640 medium (Catalog number 11875101) were used to construct environments for anchorage-independent growth of C666-1 cells. EVOSTM M5000 Imaging System was used to observe spheroids of C666-1 cells in agarose layers. Spheroids were cultured for 1 month and then stained with 0.1% crystal violet (Catalog number, V5265-2; Sigma). The Biorad ChemiDoc MP Imaging System was used to scan spheroids on 6-well plates and generate TIFF files for quantitative analysis. ImageJ program (version 1.54g) was used to determine the number of spheroids on six-well plates. Student’s *t* test was used to identify numeric differences of spheroids between control and treatments using Prism-GraphPad (Version 10). *P* value was utilized to measure significance of differences between control and treatments.

### Western blot

Total protein was extracted from cells using radioimmunoprecipitation assay buffer (RIPA; Thermo Fisher Scientific) supplemented with proteinase inhibitor (Roche cOmplete Mini Proteinase Inhibitor Cocktail Tablets). Goat anti-mouse or rabbit IgG conjugated with horseradish peroxidase (HRP) (Catalog number, 5178-2504 or 644005; Bio-Rad) was used to amplify intensities of blots. Immobilon Crescendo Western HRP substrate (WBLUR0500, Millipore) and Biorad ChemiDoc MP Imaging System were employed for detection of immunoblot on 0.2 µm nitrocellulose membrane (Catalog number, 1620112; Bio-Rad). Western blots were probed with affinity-purified rabbit antibody against EBNA1 generated in-house, or commercial antibodies to POU2F1 (Catalog number, ab272867; Abcam), CDC7 (Catalog number, sc-56275; Santa Cruz Biotechnology), and BZLF1 (Catalog number, sc-53904; Santa Cruz Biotechnology).

### RT-qPCR

Total RNAs were extracted from PDX C15, SNU719, and C666-1 cells using QIAGEN RNeasy Mini Kit (Catalog number, 74104), which were treated with VK1727 or DMSO. Two micrograms of total RNA was used to synthesize cDNA using SuperScript IV First-Strand Synthesis System (Catalog number, 18091300). The cDNA products were diluted 10 times for quantitative PCR analysis. Power SYBR Green PCR Master Mix (Catalog number 4368708) was used for quantitative PCR assay. The qPCR was performed with the provided program (95°C for 30 s; 45 cycles: 95°C for 30 s, 60°C for 10 s, 72°C for 10 s; 65°C to 95°C at 0.1°C/s for melting curve analysis) in QuantStudio 6 and 7 Pro Real-Time PCR Systems. *GUSB* was utilized as a reference gene for normalization. The relative fold change value was calculated by the 2^−ΔΔCt^ Method. ΔCt = Ct (target gene) − Ct (*GUSB*), and ΔΔCt = ΔCt (Control) − ΔCt (treatment). All RT-qPCR primers are listed in Table S6.

### Flow cytometry for detection of cell cycle

Flow cytometry for measuring the cell cycle of EBV^+^ and EBV^−^ epithelial cancer cells used PI-based cell cycle detection as previously described (37). FlowJo (version) was utilized to identify G1-S or G2-M transition events of EBV^+^ and EBV^−^ epithelial cancer cells under VK1727 and simurosertib treatments. Then Student’s *t* test was used to identify numeric differences of these events between control and treatments using Prism-GraphPad (Version 10). *P* value was employed to measure significance of differences between control and treatments.

### Relative qPCR assay for the identification of EBV copy number

Relative DNA qPCR assay was utilized to determine copy number of EBV episomes in EBV^+^ epithelial cancer cells (SNU719 and C666-1) using previously described methods (30). Briefly, genome DNAs were extracted from treated SNU719 and C666-1 cells using QIAamp DNA Blood Mini Kit (Catalog number, 51104; Qiagen). An amount of 30 ng DNA from each sample was sonicated into short size (200–600 bp) using Bioruptor Plus sonication device (Catalog number, B01020014). Then, sonicated DNA was utilized for detection of EBV copy number. EBV oriLyt (30) and GAPDH promoter regions (94) were employed to determine copy number of EBV episomes using identified primers. Relative EBV copy number was calculated using 2^−ΔCT^. The ΔCT was generated through differences of CT value between EBV OriLytic and GAPDH promoter. Then, Student’s T test was used to identify differences of EBV copy number between control and treatments using Prism-GraphPad (Version 10). *P* value was employed to measure difference significance between control and treatments.

## Data Availability

RNA-seq raw data sets (FASTQ files) are deposited in the Gene Expression Omnibus database in NCBI. The accession numbers for deposited data sets are GSE315867 (RNA-seq data sets) and GSE315844 (ChIP-seq data sets).

## SUPPLEMENTAL MATERIAL

Supplemental material mbio.00329-26-s0001.pdf Supplemental tables, figures, and legends.

Data Table S1 mbio.00329-26-s0002.xlsx RNA-seq analysis of differentially expressed genes.

Data Table S2 mbio.00329-26-s0003.xlsx Annotation of EBNA1 binding sites.

ASM does not own the copyrights to Supplemental Material that may be linked to, or accessed through, an article. The authors have granted ASM a non-exclusive, world-wide license to publish the Supplemental Material files. Please contact the corresponding author directly for reuse.
