# Supplementary material for: EBNA1 inhibitors reveal CDC7 and POU2F1 as direct functional targets in EBV epithelial cancers
Source: mBio. 2026 May 14;17(6):e00329-26. doi: 10.1128/mbio.00329-26 (PMC13251362; doi:10.1128/mbio.00329-26)

SUPPLEMENTARY DATA TABLES AND FIGURE LEGENDS

EBNA1 INHIBITORS REVEAL CDC7 AND POU2F1 AS DIRECT FUNCTIONAL TARGETS IN EBV EPITHELIAL CANCERS

Songtao He[1], Niseno Terhuja[1], Samantha S. Soldan[1], Christopher Chen[1], Joel Cassel[1], Xiangfan Yin[1], Qin Liu[1], Sun Sook Chung[1], Leonardo Josué Castro-Muñoz[1], Leena Yoon[1], Jie Wang[1], Joseph M. Salvino[1], Benjamin E. Gewurz[2], Italo Tempera[1], Troy E. Messick[1], and Paul M. Lieberman*[1]

[1] The Wistar Institute, Philadelphia, PA 19104

[2] Brigham and Women's Hospital, Harvard Medical School, Boston, MA

*Corresponding Author

Paul M. Lieberman

The Wistar Institute, Philadelphia, PA 19104

Email: lieberman@wistar.org

Phone: 215-898-9491

Running title: Gene Targets of EBNA1 Inhibitors

Keywords: EBV, EBNA1, inhibitors, epithelial cancers, CDC7, POU2F1

Data Table S1. RNA-seq analysis of differentially expressed genes across four EBNA1 inhibitor treated cell models. SNU719, C666-1 and PDX-C15 cells were treated with EBNA1 inhibitor VK1727. Mutu1 was treated with EBNA1 inhibitor VK1850. RNA-seq analyzed by DESeq.

Data Table S2. Annotation of EBNA1binding sites in cellular genomes of C666-1 and SNU719 cells. ChIPseeker package in R program were employed to annotate EBNA1 binding sites in cellular genomes of C666-1 and SNU719 cells. Bedtools intersect with -c option was used to identify conserved (or shared)EBNA1 binding sites between C666-1 and SNU719 genomes. ChIPseeker package were employed to annotate conserved/shared EBNA1 binding sites in cellular genomes of C666-1 and SNU719 cells.

Table S1. Enrichment of 88 overlapped genes in top 10 signaling pathways. 88 overlap genes were obtained from transcriptomic analysis of three EBV+ tumor derived cell models. KEGG database (Kyoto Encyclopedia of Genes and Genomes) was employed to annotate 88 overlap genes into signaling pathways. *P* and q values (adjusted *P* value) were used to assess statistical significance of signaling pathway enrichment.

Table S2. Identification of 14 EBNA1-bound targets in 88 overlaps and enrichment of conserved motif in the center of EBNA1 binding sites. EBNA1 conservative binding sites were identified in promoter, gene body or distal intergenic regions of 14 differentially expressed genes in 88 overlaps across three tumor derived cell models. Fold change denotes Log2 fold change. Annotation denotes localization of EBNA1 consensus binding site motif (shown below using Homer) in cellular genomes. TSS denotes transcription start site.

Table S3. Identification of top 16 EBNA1-bound targets specific to SNU719 cells. EBNA1 binding sites were enriched in promoter, gene body and distal intergenic regions of 69 upregulated genes particular to SNU719 cells. Fold change denotes Log2 fold change. Annotation denotes localization of EBNA1 binding sites in cellular genomes of SNU719 cells. TSS denotes transcription start site.

Table S4. Identification of top 12 EBNA1-bound targets specific to C666-1 cells. EBNA1 binding sites were enriched in C666-1 in promoter, gene body and distal intergenic regions of 28 downregulated genes exclusive to C666-1 cells. Fold change denotes Log2 fold change. Annotation denotes localization of EBNA1 binding sites in cellular genomes of C666-1 cells. TSS denotes transcription start site.

Table S5. Identification of top 12 EBNA1-bound targets specific to C15 cells. EBNA1 binding sites were enriched in promoter, gene body and distal intergenic regions of 28 downregulated genes exclusive to C15 cells. Fold change denotes Log2 fold change. Annotation denotes localization of EBNA1 binding sites in cellular genomes of C666-1 cells. TSS denotes transcription start site.

Table S6. oligonucleotide (oligo) sequences used in this study.

Figure S1. Functional analysis of VK1727 treated C15. A) Function analysis of differentially expressed genes in VK1727 treated C15. The clusterProfiler package was employed to annotate differentially expressed genes into KEGG (Kyoto Encyclopedia of Genes and Genomes) database. **B)** Enrichment scores of top 10 signaling pathways in functional analysis. **C)** Network of enriched genes in cell cycle, DNA replication and P53 signaling pathways.

Figure S2. Functional analysis of VK1727 treated C666-1. A) Function analysis of differentially expressed genes in VK1727 treated C666-1. The clusterProfiler package was employed to annotate differentially expressed genes into KEGG (Kyoto Encyclopedia of Genes

and Genomes) database. **B)** Enrichment scores of top 10 signaling pathways in functional analysis. **C)** Network of enriched genes in cell cycle and P53 signaling pathways.

Figure S3. Functional analysis of VK1727 treated SNU719. A) Function analysis of differentially expressed genes in VK1727 treated SNU719. The clusterProfiler package was employed to annotate differentially expressed genes into KEGG (Kyoto Encyclopedia of Genes and Genomes) database. **B)** Enrichment scores of top 10 signaling pathways in functional analysis. **C)** Network of enriched genes in cell cycle and P53 signaling pathways.

Figure S4. Overlaps of differentially expressed genes across three VK1727 treated tumor derived cell models. A) Overlaps of upregulated genes across VK1727 treated C15, C666-1 and SNU719 cells. Three-way Venn diagram illustrated 35 overlaps of upregulated genes across three VK1727 treated tumor derived cell models. **B)** Overlaps of downregulated genes across VK1727 treated C15, C666-1 and SNU719 cells. Three-way Venn diagram illustrated 53 overlaps of downregulated genes across three VK1727 treated tumor derived cell models.

Figure S5. Transcriptomic analysis of EBV genes after VK1727 treatments. Heatmap of differential expression for EBV genes after VK1727 treatments in PDX-15 (left column), C666-1 (middle column), or SNU719 (right column). Only genes with padj<0.1 were considered. Statistical comparisons between means were performed using DeSeq-2 package in R program. *: p value <0.05, **: p value <0.01, ***: p value <0.005, ****: p value <0.001.

Figure S6. Integrated ChIP and RNA-seq analysis highlighting EBNA-bound targets exclusive to SNU719 cells. A) Comparative analysis of EBNA1 ChIP-seq datasets between SNU719 and C666-1 cells. Two-way Venn diagram showed EBNA1 binding sites identified in promoter, gene body and distal intergenic regions of 472 genes specific to SNU719 cells. **B)** Integrated analysis identified EBNA1 binding sites in promoter, gene body and distal intergenic

regions of 69 upregulated genes particular to SNU719 cells. **C)** Integrated analysis identified EBNA1 binding sites in promoter, gene body and distal intergenic regions of 28 downregulated genes particular to SNU719 cells.

Figure S7. Integrated ChIP and RNA-seq analysis highlighting EBNA-bound targets exclusive to C666-1 cells. A) Comparative analysis of EBNA1 ChIP-seq datasets between SNU719 and C666-1 cells. Two-way Venn diagram showed EBNA1 binding sites identified in promoter, gene body and distal intergenic regions of 472 genes specific to SNU719 cells. **B)** Integrated analysis identified EBNA1 binding sites in promoter, gene body and distal intergenic regions of 15 upregulated genes particular to C666-1 cells. **C)** Integrated analysis identified EBNA1 binding sites in promoter, gene body and distal intergenic regions of 10 downregulated genes particular to C666-1 cells.

Figure S8. Integrated ChIP and RNA-seq analysis highlighting EBNA-bound targets exclusive to C15. A) Comparative analysis of EBNA1 ChIP-seq datasets between SNU719 and C666-1 cells. Two-way Venn diagram showed EBNA1 binding sites identified in promoter, gene body and distal intergenic regions of 472 genes specific to SNU719 cells. **B)** Integrated analysis identified EBNA1 binding sites in promoter, gene body and distal intergenic regions of 4 upregulated genes particular to C15 cells. **C)** Integrated analysis identified EBNA1 binding sites in promoter, gene body and distal intergenic regions of 7 downregulated genes particular to C15 cells.

Figure S9. ChIP-qPCR validation of VK1727 reduced EBNA1 binding to DS and Qp. A-I) ChIP-qPCR of IgG or EBNA1 binding to **A**) DS in PDX C15, **B)** DS region in C666-1 **C)** DS in SNU719, **D)** Qp in PDX C15, **E)** Qp in C666-1, **F)** Qp in SNU719, **G)** oriLyt in PDX C15, **H)** oriLyt in C666-1, and **I)** oriLyt in SNU719. Error bars represent mean + SEM. Statistical comparisons between means were performed by Student's t-test (2-tailed). *: p value <0.05, **: p value <0.01, ***: p value <0.005, ****: p value <0.001. Y-axis numerical scale labels % Input denotes ChIP-qPCR yield % input

Figure S10. RT-qPCR assay and western blot detect transcription of CDC7 and POU2F1 in HK-1 cells. **A)** RT-qPCR assay used to detect transcription of CDC7, POU2F1, CREB5 and ATF3 in HK-1 (EBV negative) cells. **B)** Western blot analysis of HK-1 cells transfected with pCMV-FLAG empty vector (EV) or FLAG-EBNA1 or EBNA1-DC probed for FLAG (top panel), POU2F1, CDC7, or β-Actin. Transfected HK-1 cells were collected for western blot analysis after 48-hour hygromycin B selection.

Figure S11. ChIP-qPCR assay identify multiple functions of POU2F1 in SNU719 cells.

A-D) ChIP-qPCR assay for EBNA1, H3K27ac, H3K27me3 or IgG control binding to viral (FR, DS and Qp) and cellular (CDC7 promoter) genomes in control and POU2F1 knockdown SNU719 cells.

Figure S12. Comparative analysis of transcription of POU2F1 and POU2F2 across four EBNA1 inhibitor treatments. **A)** Comparative analysis of POU2F1(Oct-1) and POU2F2 (Oct-2) in RNA-seq dataset of VK1727 treated C15. **B-D)** Comparative analysis of POU2F1 and POU2F2 in RNA-seq dataset of VK1727 treated C666-1(B), SNU719(C) and VK1850 treated MutuI(D). Violin plots illustrated expression differentiation of POU2F1 and POU2F2 after EBNA1 inhibitor treatments. FPKM denotes fragments *Per* Kilobase of transcript per Million mapped reads. Error bars represent mean + SEM. Statistical comparisons between means were performed by Student's t-test (2-tailed). *: p value <0.05, **: p value <0.01, ***: p value <0.005, ****: p value <0.001.

Figure S13. Sphere formation assay detect potentiality of POU2F1 in maintaining cancer stem cell property of C666-1 cells. **A)** Soft Agar assay showed VK1727 treatments reduced spheroid expansion of C666-1 cells in 3D culture. **B)** Soft Agar assay illustrated POU2F1 knockdown impaired spheroid growth of C666-1 cells in 3D culture.

Figure S14. Resazurin assay and colony formation detect selectively inhibitory effects of CDC7 inhibitors on growth of EBV+ epithelial cancer cells. A-B) Resazurin assay used to determine the selective dose of CDC7 inhibitor Simurosertib for inhibiting growth of (**A**) SNU719 cells relative to AGS at 100 nM, 500 nM, 1 μM, 1.5 μM and 2 μM of Simurosertib, or (B) C666-1 relative to HK-1 at 1 μM, 1.5 μM, 5 μM, 7 μM and 10 μM. Inhibition percentage of cell growth were compared using Student's t-test (2-tailed: (amend as required) *: p value <0.05, **: p value <0.01, ***: p value <0.005, ****: p value <0.001. **C-D)** Colony formation assay detects selective effects of Simurosertib on proliferation of C666-1 cells. VK1727 treatments were used as positive control. Error bars represent mean + SEM. Statistical comparisons between means were performed by Student's t-test (2-tailed: (amend as required) *: p value <0.05, **: p value <0.01, ***: p value <0.005, ****: p value <0.001.

Figure S15. Comparative analysis of RNA-seq datasets across four EBNA1 inhibitor treatments. A–B) Shared and Distinct Gene Regulation. 4-way Venn diagrams illustrating the intersection of up-regulated (A) and down-regulated (B) genes across SNU719, C15, C666-1 treated VK-1727 and MutuI treated VK1850. Intersections are based on differentially expressed genes identified at $p < 0.05$. **C)** Functional analysis of overlaps of upregulated and downregulated genes identified top 10 signaling pathways ($p < 0.05$) in KEGG database. **D)** Western blots detect expression of POU2F1 and CDC7 in VK1727 treated MutuI (EBV positive) and BJAB (EBV negative) cells.

Table S1. Enrichment of 88 overlapped genes in top 10 signaling pathways

term	p-value	q-value	overlap_genes
CELL CYCLE	3.025996e-08	0.000005	[CDKN2B, MCM7, CCND1, CDCA5, CDK1, MCM3, CDC7, MCM5, CDC25C]
P53 SIGNALING PATHWAY	3.351372e-04	0.015442	[RRM2, CCND1, ZMAT3, CDK1]
HUMAN CYTOMEGALOVIRUS INFECTION	4.547861e-04	0.015442	[PTGER4, CXCL8, PPP3CC, CCND1, CALR, IL6R]
DNA REPLICATION	4.865391e-04	0.015442	[MCM7, MCM3, MCM5]
NON-ALCOHOLIC FATTY LIVER DISEASE	5.942555e-04	0.015442	[ERN1, SREBF1, CXCL8, DDIT3, IL6R]
CELLULAR SENESCENCE	6.300573e-04	0.015442	[CDKN2B, CXCL8, PPP3CC, CCND1, CDK1]
MICRORNAS IN CANCER	7.064888e-04	0.015442	[ZEB1, CCND1, CDCA5, CDC25C, CD44]
PATHWAYS IN CANCER	2.185511e-03	0.041798	[DLL4, PTGER4, CDKN2B, CXCL8, CCND1, CCDC6, IL6R, LMNB2]
LIPID AND ATHEROSCLEROSIS	2.554063e-03	0.043419	[ERN1, POU2F1, CXCL8, PPP3CC, DDIT3]
SPLICEOSOME	3.764733e-03	0.057600	[ALYREF, THOC3, LSM4, SNRPB]

Table S2. Identification of 14 EBNA1-bound targets in 88 overlaps and enrichment of conserved motif in the center of EBNA1 binding sites

Gene	EBNA1-ChIP-Seq		RNA-seq					
	annotation	distance To TSS (bp)	Fold Change (PDX-C15)	P-ajusted (PDX-C15)	Fold Change (C666-1)	P-ajusted (C666-1)	Fold Change (SNU719)	P-ajusted (SNU719)
**CDC7**	Promoter (<=1kb)	-289	-1.976291567	2.32E-06	-1.758070746	5.37E-57	-1.457376215	2.10E-09
**POU2F1**	Intron (intron 1 of 11)	23335	-1.719983892	6.27E-06	-2.017117692	2.90E-71	-1.036441553	1.66E-19
**SOX2**	Distal Intergenic	187294	-1.433346696	3.16E-22	-1.551253873	1.27E-70	-1.234757988	0.041669533
**IL6R**	Promoter (<=1kb)	-350	-0.656859605	0.049948999	-1.454477458	0.041908303	-1.428420297	1.56E-52
**NDOR1**	Promoter (1-2kb)	1399	-0.609983983	0.005889939	-0.54901116	0.007686957	-1.063147734	0.007140661
**PTPRM**	Intron (intron 2 of 30)	-91795	0.220615164	0.046224963	0.620893429	6.21E-11	2.647715371	1.26E-38
**CCDC6**	Promoter (2-3kb)	-2048	0.593773505	0.029046292	0.579970653	1.70E-06	0.5922023	0.04100249
**PPP3CC**	Promoter (<=1kb)	153	0.609031434	0.007175527	0.87626514	0.028591984	0.79934114	4.79E-11
**PAK3**	Promoter (<=1kb)	605	0.631194938	0.008345074	2.210059312	0.009625458	0.965879982	6.61E-23
**TMCC1**	Promoter (1-2kb)	-1835	0.6503836	0.028150434	0.85655843	0.005611837	0.765879982	6.61E-23
**SOX6**	Intron (intron 8 of 14)	316658	0.841055671	0.04610885	1.262230909	0.048518941	1.028638997	0.009715279
**ATF3**	Distal Intergenic	-19608	1.436413618	0.017178537	1.528558872	1.89E-75	1.221031487	8.18E-06
**SOX9**	Distal Intergenic	-273002	-0.460500207	0.000372843	-0.424958075	6.29E-06	-0.581602255	9.39E-06
**DEPDC1**	Distal Intergenic	-103030	-0.319642518	0.028410639	-0.607596572	9.96E-05	-0.799968101	5.36E-21

Rank	Motif	Name	P-value	log P-pvalue	q-value (Benjamini)
1	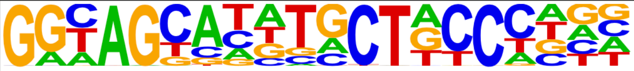	EBNA1(EBV-virus)/Raji-EBNA1-ChIP-Seq(GSE30709)/Homer	1e-245	-5.653e+02	0.0000

Table S3. Identification of top 16 EBNA1-bound targets specific to SNU719 cells.

Gene	RNA-seq		EBNA1-ChIP-seq	
	Fold Change	P adjusted value	annotation	distance to TSS
**NBEA**	4.162129548	2.26E-05	Intron (intron 37 of 56)	-77260
**EDARADD**	4.008757262	0.004253585	Intron (intron 2 of 5)	18379
**TTTY13**	1.524623981	0.043523234	Distal Intergenic	-30616
**CDH11**	1.524073605	0.03321564	Intron (intron 4 of 8)	14639
**B3GAT2**	1.418789424	0.005137916	Intron (intron 1 of 3)	52049
**MB21D2**	1.149096869	1.34E-32	Distal Intergenic	-137300
**DST**	1.062261676	1.61E-31	Intron (intron 8 of 93)	-6430
**CD83**	-1.0202638	2.39E-14	Distal Intergenic	431592
**SLC14A1**	-1.0479144	2.83E-18	Distal Intergenic	-14006
**ITGB8**	-1.061531	1.34E-21	Intron (intron 2 of 14)	7558
**SLITRK6**	-1.1459252	1.64E-38	Distal Intergenic	148231
**FABP3**	-1.1756315	0.00718457	Distal Intergenic	-16770
**RHD**	-1.2841348	0.0109992	Intron (, intron 3 of 6)	25323
**ADAM11**	-1.5722998	1.64E-06	Promoter (1-2kb)	-1499
**LINC01091**	-1.7584772	3.67E-19	Distal Intergenic	286189
**DOCK8**	-2.5121914	7.93E-12	5' UTR	43699

Table S4. Identification of top 12 EBNA1-bound targets specific to C666-1 cells.

Gene	RNA-seq		EBNA1-ChIP-seq	
	Fold Change	P adjusted value	annotation	distance to TSS
**CPM**	2.14822628	2.15E-12	Intron (intron 2 of 8)	10642
**PURPL**	1.13966762	1.19E-06	Distal Intergenic	680118
**PLCL2**	0.7579953	8.47E-14	Promoter (<=1kb)	488
**GABRG3**	0.7264053	6.18E-12	Intron (intron 2 of 5)	30912
**AMOTL2**	0.53987401	3.49E-10	Distal Intergenic	54202
**DUSP5**	0.51533681	1.68E-08	Distal Intergenic	-6980
**AVIL**	0.51237141	0.01567511	Promoter (<=1kb)	-353
**PGK1**	-0.5160254	5.06E-08	Promoter (<=1kb)	0
**MSMO1**	-0.6094362	0.025851	Promoter (2-3kb)	-2025
**PEG10**	-0.7364807	1.73E-15	Distal Intergenic	59108
**GRM4**	-1.2555305	2.05E-09	Intron (intron 1 of 8)	13329
**XIRP1**	-1.4248876	0.00564262	Distal Intergenic	-6400

Table S5. Identification of 11 EBNA1-bound targets specific to C15 cells.

Gene	RNA-seq		EBNA1-ChIP-seq	
	Fold Change	P adjusted value	annotation	distance to TSS
**MATN1-AS1**	0.588224564	0.032890243	Distal Intergenic	-622222
**TBXAS1**	0.348169924	0.029720162	Promoter (<=1kb)	483
**HERC1**	0.319350831	0.004621564	Intron (intron 1 of 77)	7538
**NAV2**	0.306842667	0.019115487	Intron (intron 20 of 37)	11667
**CEP135**	-0.282589237	0.020848134	Intron (intron 12 of 25)	15580
**PGK1**	-0.289230492	0.007410798	Promoter (<=1kb)	0
**OGFRL1**	-0.389007753	0.006724158	Distal Intergenic	-10336
**MSMO1**	-0.516116586	1.21E-06	Promoter (2-3kb)	-2025
**PABPC4L**	-0.712189783	1.79E-05	Distal Intergenic	427326
**CD6**	-0.727342482	0.021834543	Exon (exon 5 of 12)	37949
**PWWP3B**	-1.030678778	0.038300536	Intron (intron 2 of 3)	28542

Table S6. oligonucleotide (oligo) sequences used in this study

Oligo	Experiment	sequence
**CDC7 Forward**	EBNA1-ChIP-qPCR	AACCCACCTACCTCATAGCC
**CDC7 Reverse**	EBNA1-ChIP-qPCR	TTCCTTTTCGTTGAGTGCCC
**POU2F1 Forward**	EBNA1-ChIP-qPCR	GCCAAGCTCACTCACACAG
**POU2F1Reverse**	EBNA1-ChIP-qPCR	TCTGGCTCTTCTCATGTGCT
**CDC7 Forward**	Pol II-pS5 ChIP -qPCR	GTTTCCGACGGTTTGTTCCA
**CDC7 Reverse**	Pol II-pS5 ChIP -qPCR	AGAGACCGAACCAGATGCTT
**POU2F1 Forward**	Pol II-pS5 ChIP -qPCR	CAGAGCGAGGGAGGGTTTAT
**POU2F1Reverse**	Pol II-pS5 ChIP -qPCR	AGCCGGGGTTGA GTATGAAT
**POU2F1-1**	shRNA-1	CCGGGCAAAGGAGAGAAGGGAGAAACTCGAGTTTCTCCCTTCTCTCCTTTGCTTTTT
**POU2F1-2**	shRNA-2	CCGGCCAAACTACCATCTCTCGATTCTCGAGAATCGAGAGATGGTAGTTTGGTTTTT
**POU2F1-3**	shRNA-3	CCGGGCTGTGACGAATCTTTCAGTTCTCGAGAACTGAAAGATTCGTCACAGCTTTTT
**CDC7 Forward**	RT-qPCR	AGTGCCTAACAGTGGCTGG
**CDC7 Reverse**	RT-qPCR	CACGGTGAACAATACCAAACTGA
**POU2F1 Forward**	RT-qPCR	gaggagcagcgagtcaagat
**POU2F1Reverse**	RT-qPCR	ggaaagctcttcttacttttccag
**gusB Forward**	RT-qPCR	CGCCCTGCCTATCTGTATTC
**gusB Reverse**	RT-qPCR	TCCCCACAGGGAGTGTGTAG
**CREB5 Forward**	RT-qPCR	GAGCGACAAATGTCAGTGAACTCC
**CREB5 Reverse**	RT-qPCR	TGAGTCAATGCAGCCTTCAACC

Figure S1. Functional analysis of VK1727 treated C15.

A

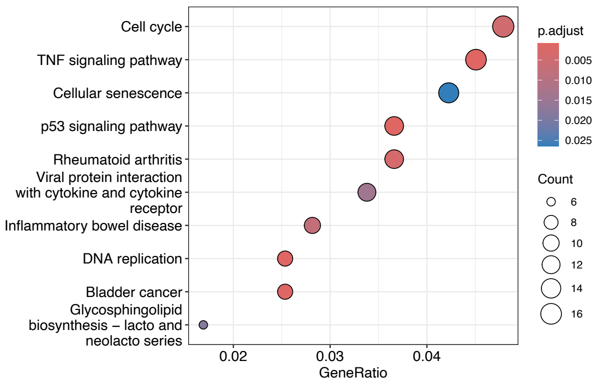

B

Description	zScore	P adjusted
**Cell cycle**	5.4132	0.0015
DNA replication	6.1683	0.0015
Biosynthesis of amino acids	5.3321	0.0025
Transcriptional misregulation in cancer	4.7436	0.0028
HIF-1 signaling pathway	4.9341	0.0028
p53 signaling pathway	4.8067	0.0054
TNF signaling pathway	4.5530	0.0054
Bladder cancer	4.8903	0.0077
Rheumatoid arthritis	4.2630	0.0120

C

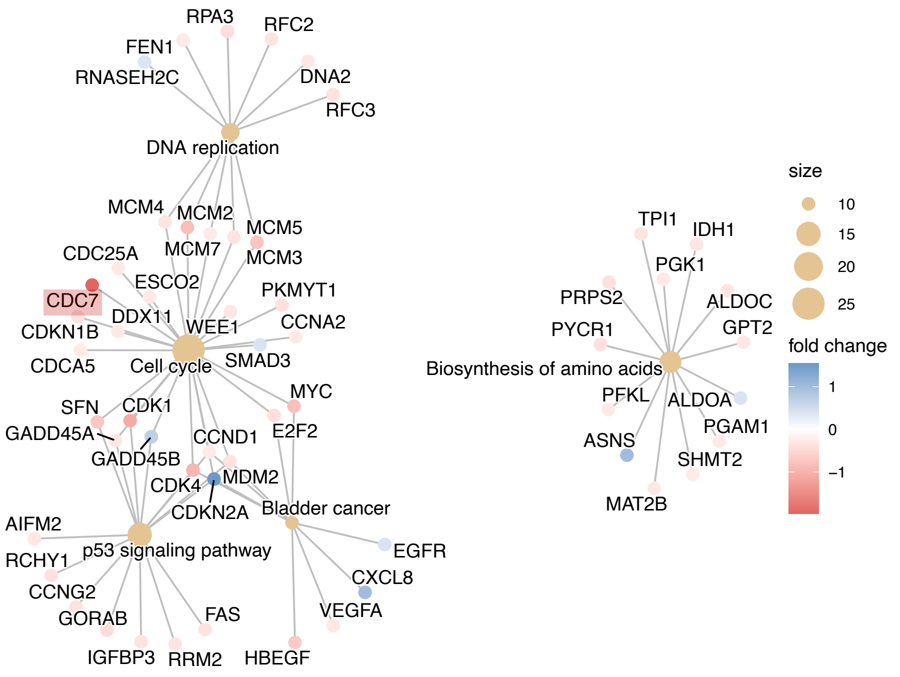

Figure S2. Functional analysis of VK1727 treated C666-1.

A

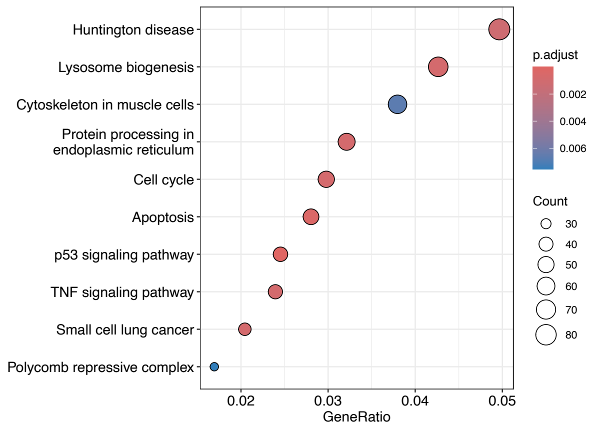

B

Description	zScore	P adjusted
p53 signaling pathway	8.4890	0.0000
Apoptosis	5.1154	0.0003
Small cell lung cancer	4.8562	0.0008
Lysosome biogenesis	4.5716	0.0008
Protein processing in endoplasmic reticulum	4.5956	0.0008
**Cell cycle**	4.5950	0.0008
TNF signaling pathway	4.5973	0.0009
Huntington disease	4.3068	0.0012
Cytoskeleton in muscle cells	3.8503	0.0067
Polycomb repressive complex	3.9471	0.0075

C

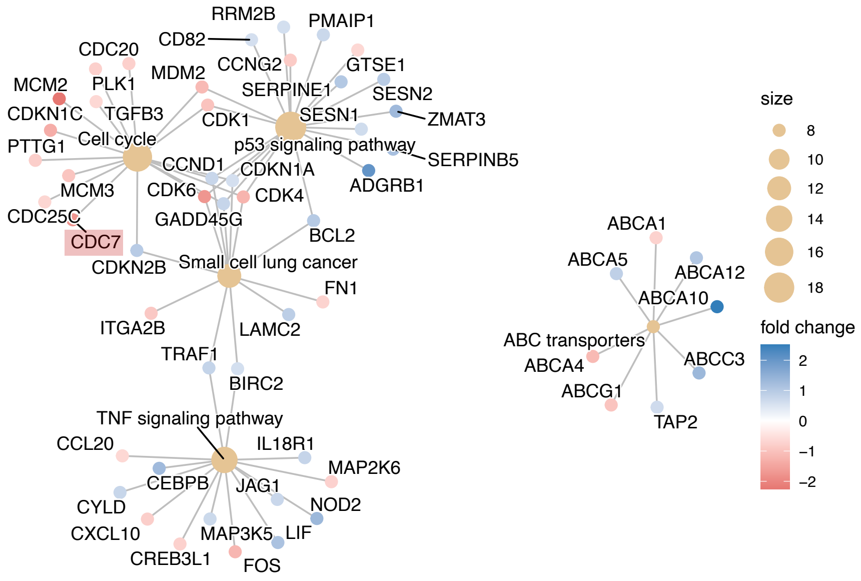

Figure S3. Functional analysis of VK1727 treated SNU719.

A

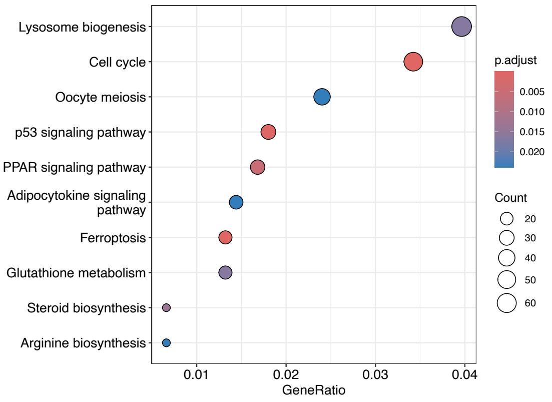

B

Description	zScore	P adjusted
**Cell cycle**	6.0708	0.0000
Ferroptosis	5.8809	0.0001
p53 signaling pathway	5.0565	0.0006
PPAR signaling pathway	4.3673	0.0051
Steroid biosynthesis	4.3601	0.0132
Glutathione metabolism	3.9343	0.0165
Lysosome biogenesis	3.6552	0.0165
Adipocytokine signaling pathway	3.6284	0.0238
Oocyte meiosis	3.4724	0.0238
Arginine biosynthesis	3.7754	0.0238

C

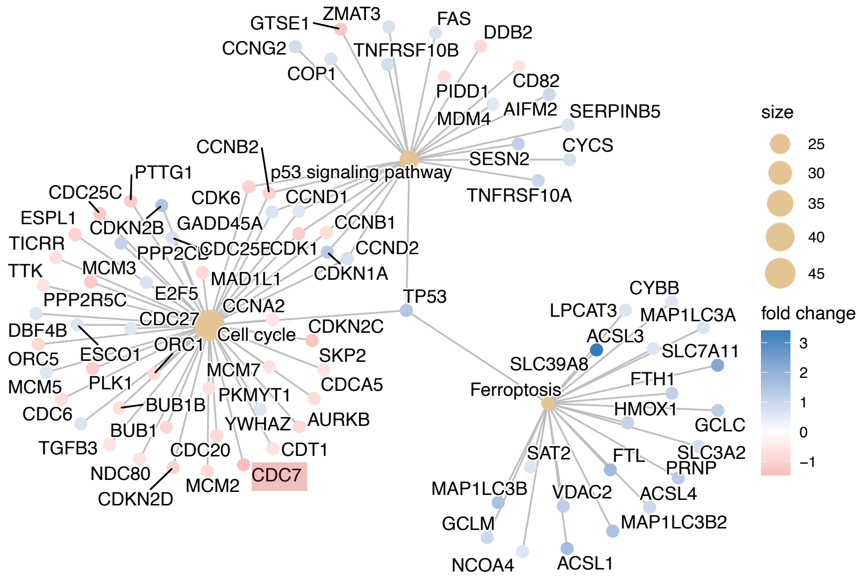

Figure S4. Overlaps of differentially expressed genes across three VK1727 treated tumor derived cell models.

A

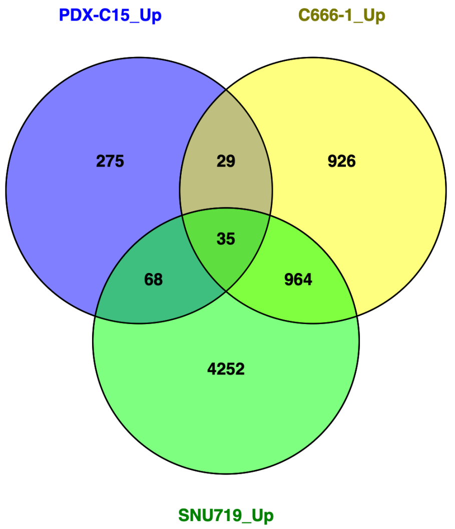

Overlaps of upregulated genes	
PTPRM	ZFAS1
AFF4	HIVEP2
PARP6	CCND1
DCXR	CCDC6
CALCOCO2	PPP3CC
ARHGEF3	PAK3
GOSR2	TMCC1
ABCC3	DDIT3
ERN1	ZMAT3
NECAP1	MXD1
ZZEF1	SOX6
NBPF1	OAS2
ARRDC3	CDKN1A
NFIL3	ZEB1
RGS2	CXCL8
NCKAP5	CDKN2B
HERPUD1	ATF3
ZBTB43	

B

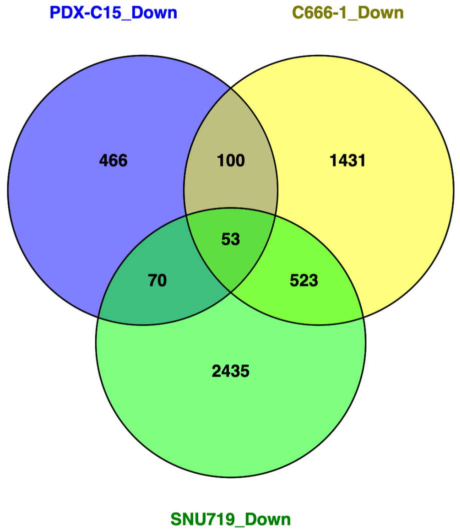

Overlaps of downregulated genes			
CDC7	ALYREF	UHRF1	JADE1
CD44	MCM5	DLL4	MYLIP
POU2F1	RRM2	H2AFZ	GPR137
SOX2	LMNB2	HIST1H4A	CALR
MYCN	SREBF1	OLMALINC	C20orf27
SLC15A3	TSPAN4	SOX9	FKBP8
CDK1	ALDOC	PIMREG	PFKL
CDC25C	WDR34	RASGEF1B	CDCA5
MCM2	DEPDC1	ANP32E	MCM7
MCM3	GAS6	THOC3	IER2
PTGER4	RASSF7	NAT14	RECQL4
IL6R	TK1	HMGN2	SNRPB
NDOR1	LSM4	PTPA	PAFAH1B3
TMC8			

Figure S5. Transcriptomic analysis of EBV genes after VK1727 treatments.

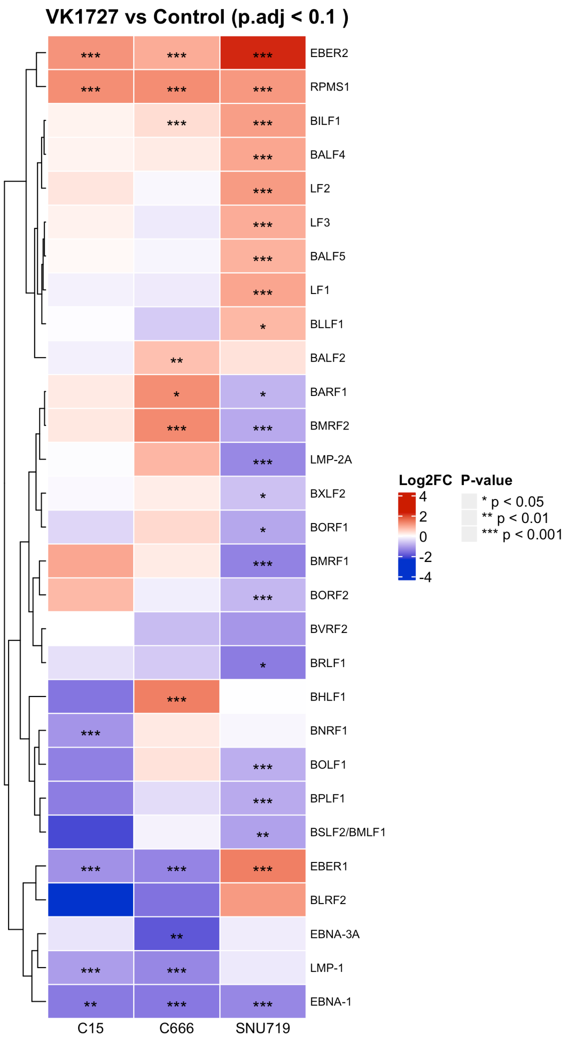

Figure S6. Integrated ChIP and RNA-seq analysis highlighting EBNA-bound targets exclusive to SNU719 cells.

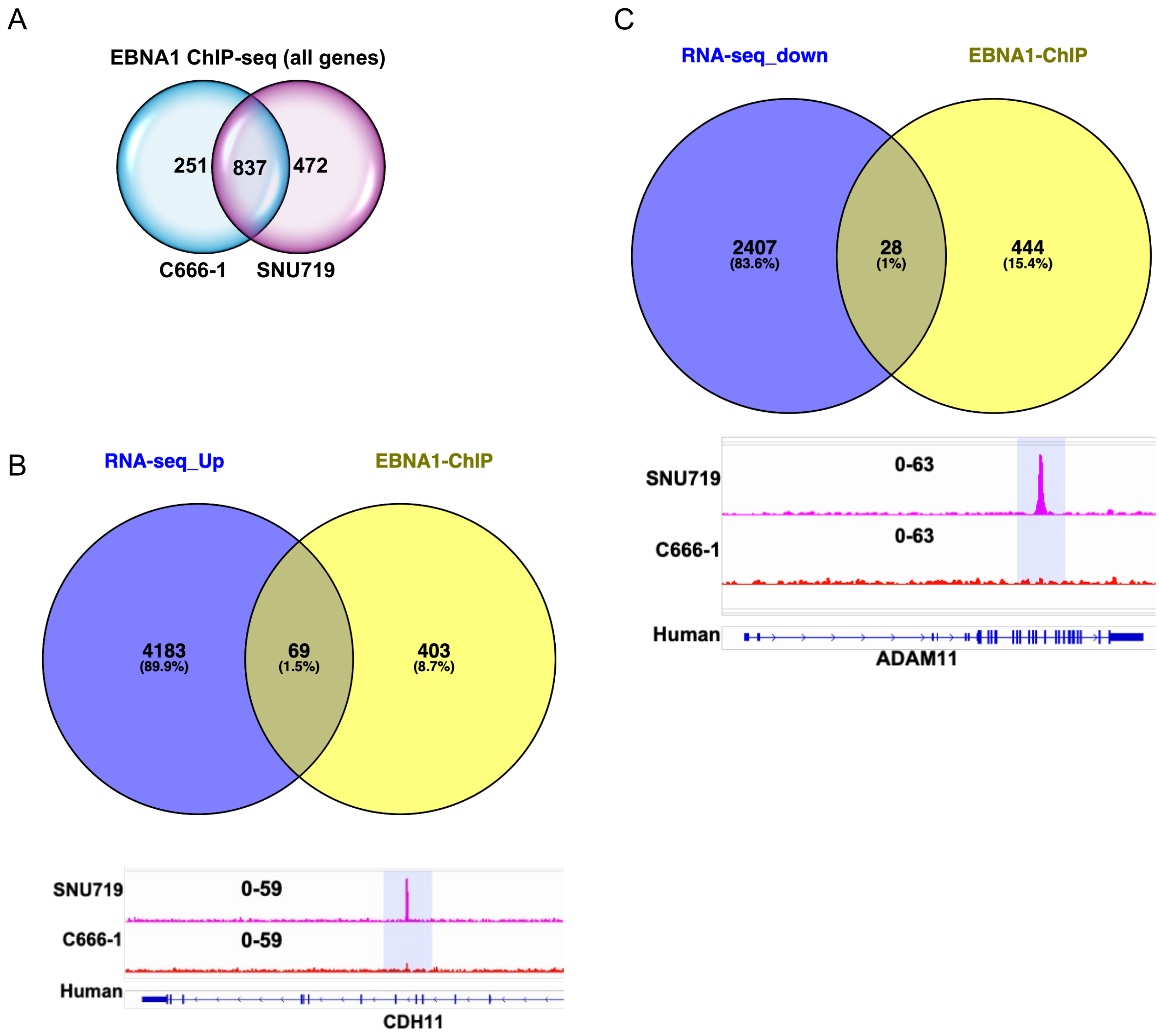

Figure S7. Integrated ChIP and RNA-seq analysis highlighting EBNA-bound targets exclusive to C666-1 cells.

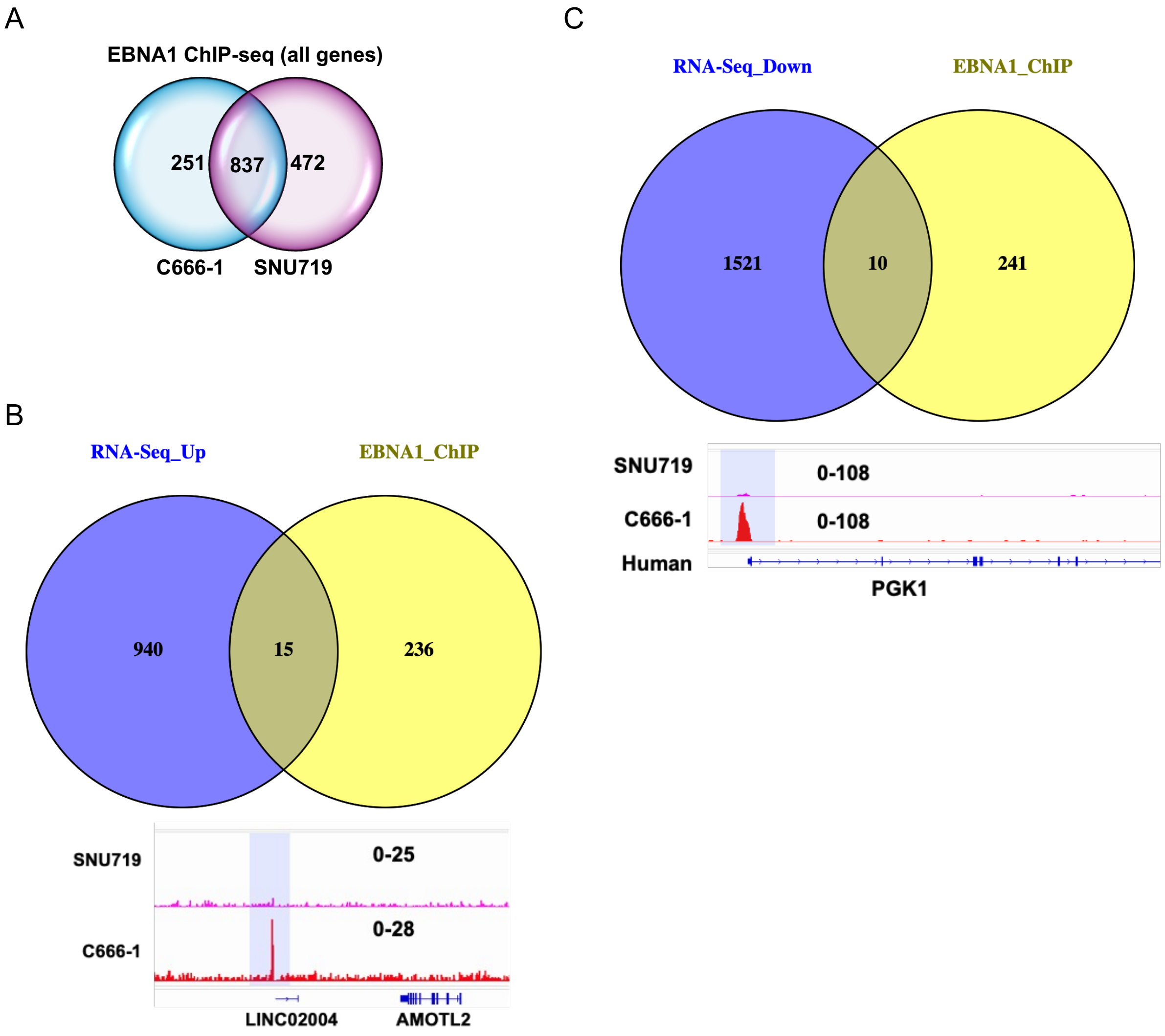

Figure S8. Integrated ChIP and RNA-seq analysis highlighting EBNA-bound targets exclusive to C15 cells.

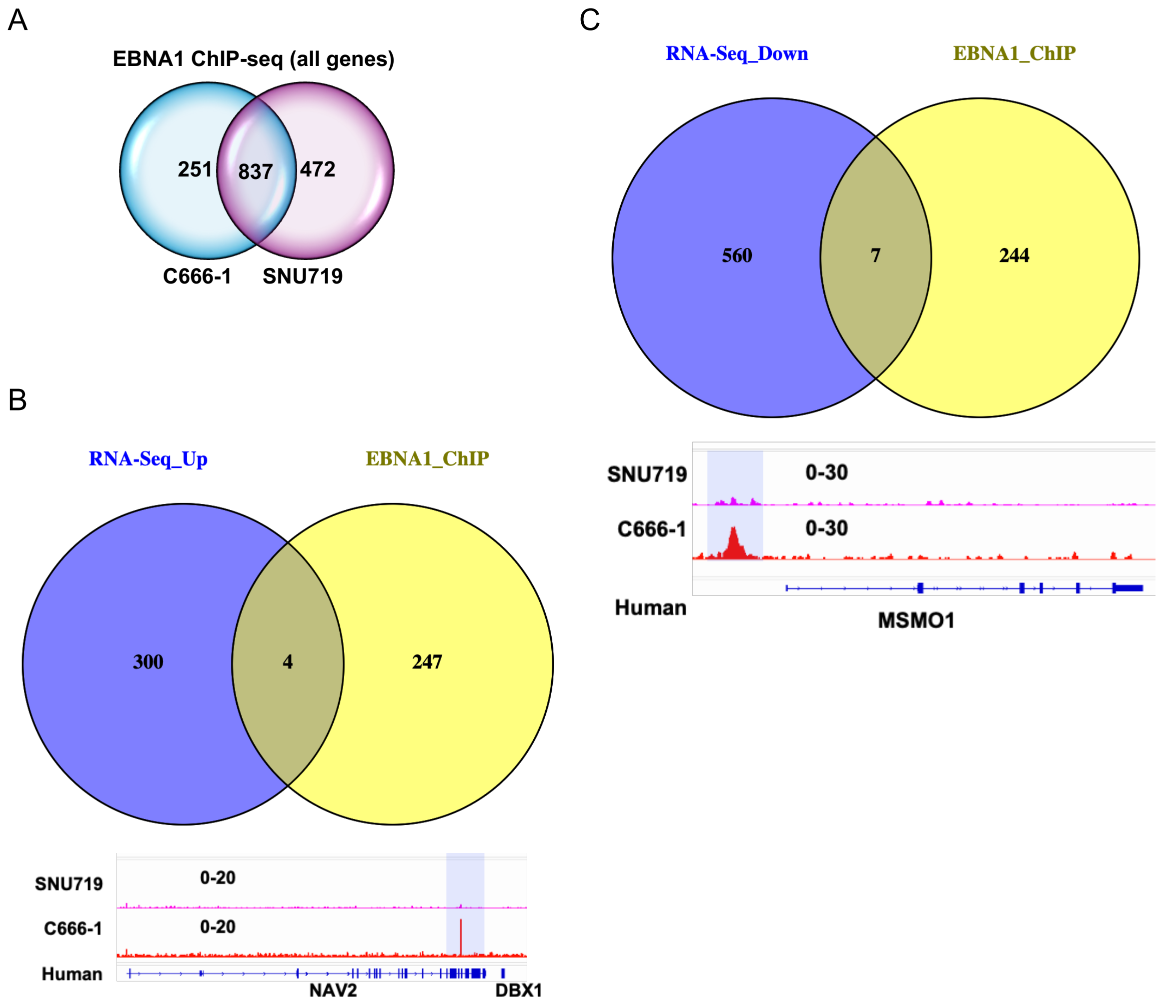

Figure S9. ChIP-qPCR validation of VK1727 reduced EBNA1 binding to DS and Qp.

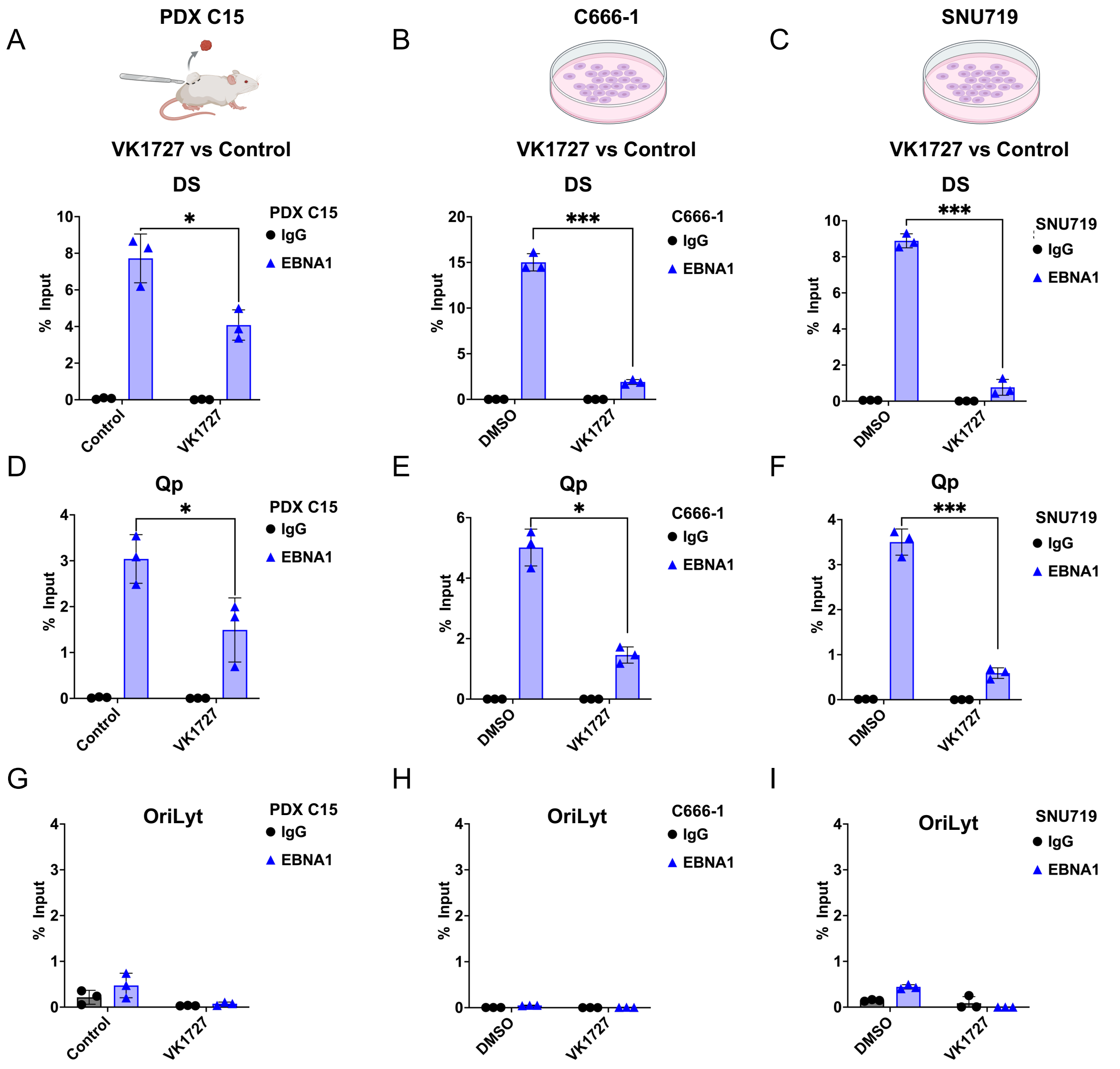

Figure S10. RT-qPCR assay and western blot detect transcription of CDC7 and POU2F1 in HK-1 cells.

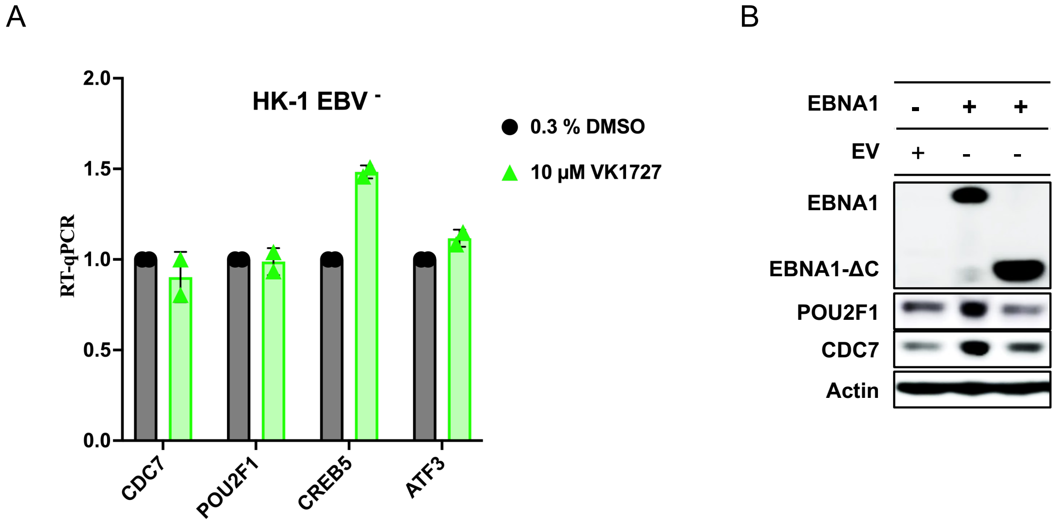

Figure S11. ChIP-qPCR assay identify multiple functions of POU2F1 in SNU719 cells.

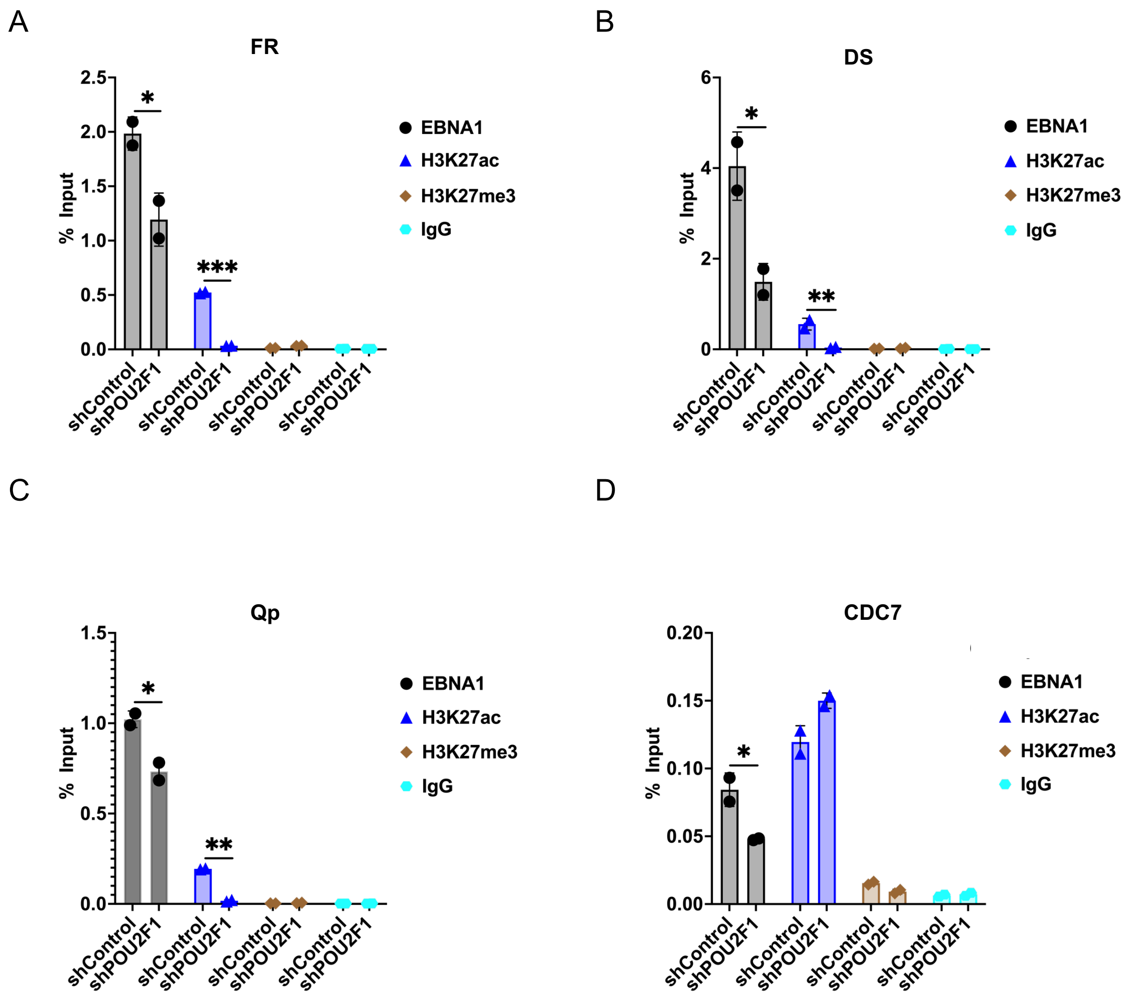

Figure S12. Comparative analysis of transcription of POU2F1 and POU2F2 across four EBNA1 inhibitor treatments.

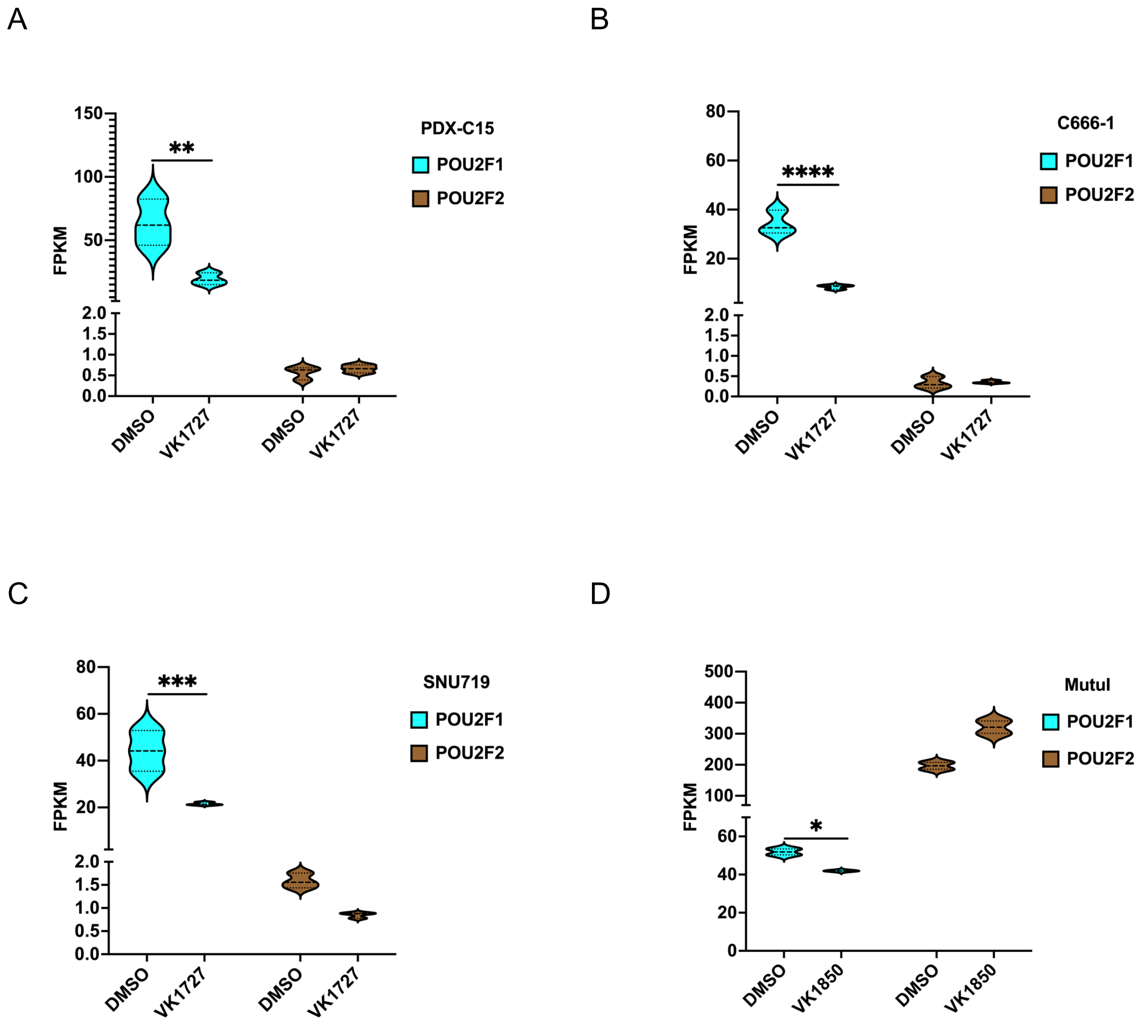

Figure S13. Sphere formation assay detect potentiality of POU2F1 in maintaining cancer stem cell property of C666-1 cells.

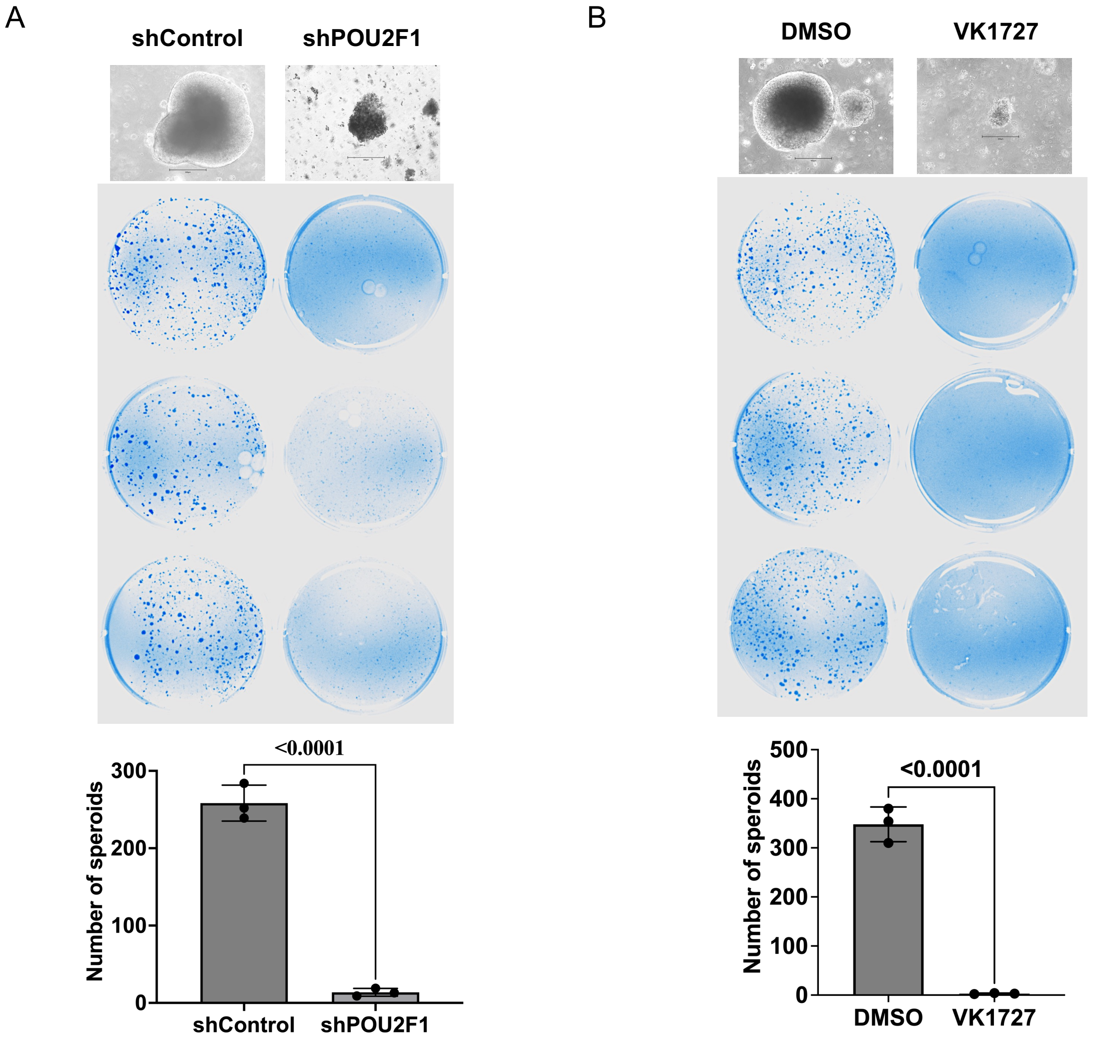

Figure S14. Resazurin assay and colony formation detect selectively inhibitory effects of CDC7 inhibitors on growth of EBV+ epithelial cancer cells.

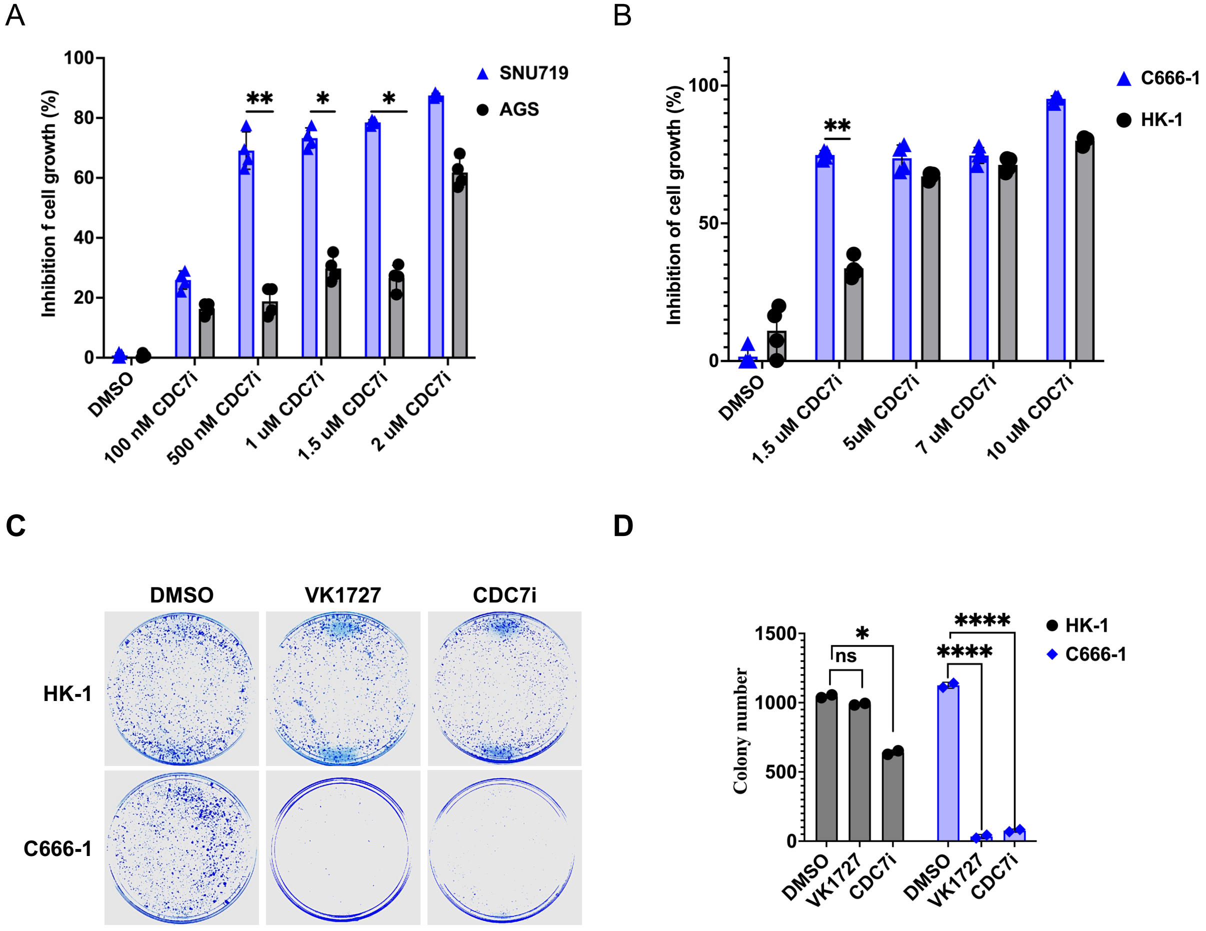

Figure S15. Comparative analysis of RNA-seq datasets across four EBNA1 inhibitor treatments.

A

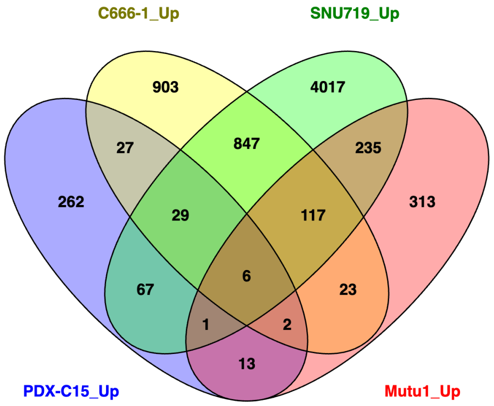

Overlaps of upregulated genes(6):
ERN1, NBPF1, ARRD3, HERPUD1, ZFAS1, CCDC6

B

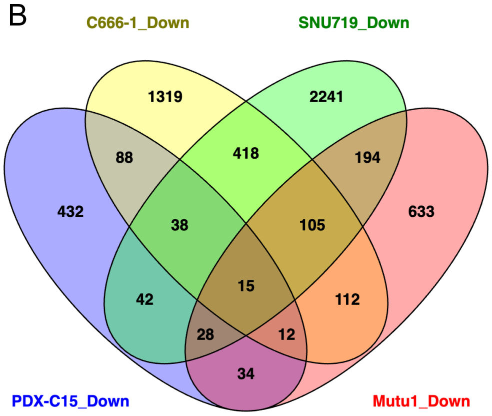

Overlaps of downregulated genes(15):
CDC7, POU2F1, SOX2, CDK1, CDC25C, MCM3, PTGER4, MCM5, PPM2, DEPDC1, TK1, LSM4, CDCA5, MCM7, SNRPB

C

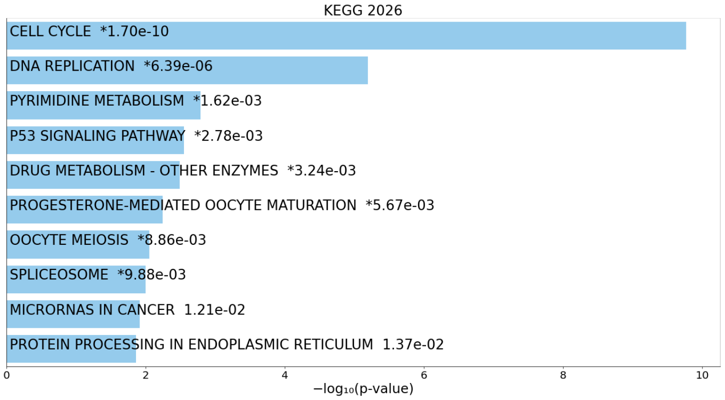

D

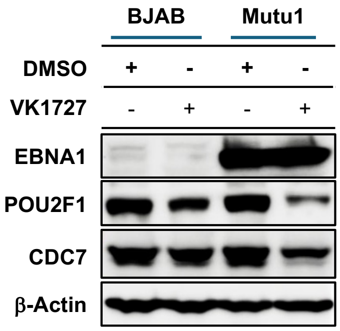

Supplement: Supplemental material — Supplemental tables, figures, and legends. [file mbio.00329-26-s0001.pdf]
